# HLA Gene Polymorphisms in Romanian Patients with Chronic Lymphocytic Leukemia

**DOI:** 10.1155/2024/8852876

**Published:** 2024-02-28

**Authors:** Maria Tizu, Bogdan Calenic, Mihai Hârza, Bogdan M. Cristea, Ion Maruntelu, Andreea M. Caragea, Adriana Talangescu, Alina Dima, Alexandra E. Constantinescu, Ileana Constantinescu

**Affiliations:** ^1^Immunology and Transplant Immunology, Carol Davila University of Medicine and Pharmacy, 258 Fundeni Avenue, Bucharest 022328, Romania; ^2^Centre of Immunogenetics and Virology, Fundeni Clinical Institute, 258 Fundeni Avenue, Bucharest 022328, Romania; ^3^Colentina Clinical Hospital, Rheumatology Department Bucharest, 19-21 Stefan Cel Mare Street, Bucharest 020125, Romania

## Abstract

**Materials and Methods:**

This study included 66 patients with CLL, diagnosed between 2020 and 2022, and 100 healthy controls. HLA class I and class II genes (HLA-A/B/C, HLA-DQA1/DQB1/DPA1/DPB1, and HLA-DRB1/3/4/5) were investigated using next-generation sequencing technology.

**Results:**

Several HLA alleles were strongly associated with CLL. The most important finding was that HLA-DRB1^*∗*^04:02:01 (*p*=0.001, OR = 1.05) and HLA-DRB3^*∗*^02:01:01 (*p*=0.009, OR = 1.03) have a predisposing role in CLL development. Moreover, we identified that HLA-A^*∗*^24:02:01 0.01 (*p*=0.01, OR = 0.38), HLA-DQA1^*∗*^05:05:01 (*p*=0.01, OR = 0.56), HLA-DQB1^*∗*^03:02:01 (*p*=0.03, OR = 0.40), and HLA-DRB4^*∗*^01:03:01 (*p*=0.03, OR = 0.54 alleles have protective roles. Correlations between HLA expression and gender showed that women had a higher expression of protective HLA alleles when compared to men.

**Conclusions:**

Our data are the first to indicate that in Romanian patients with CLL, the HLA-A^*∗*^24:02:01 and HLA-DQA1^*∗*^05:05:01 alleles have a protective role against CLL development, whereas HLA-DRB1^*∗*^04:02:01 and HLA-DRB3^*∗*^02:01:01alleles are positively associated with CLL.

## 1. Introduction

Chronic lymphocytic leukemia (CLL) has been one of the most documented lymphoproliferative disorders in the last few decades [1] and is one of the most common neoplasms in the developed world. It is characterized by clonal proliferation of B lymphocytes and their accumulation in the bone marrow, lymph nodes, and spleen [1, 2]. It is more prevalent in Western countries and has a low frequency in Asian and African-American populations [3–5]. Among the risk factors of CLL for these patients, genetic predisposition seems to have a greater impact than environment; this conclusion is supported by the aggregation of the disease within families [6]. The strong genetic component is represented by chromosomal aberrations in 80% of patients, offering valuable information about the prognosis of the disease [7]. The chromosomal abnormalities encountered most often are deletions; some are associated with a good prognosis (del (13q)), whereas others correlate with a poor prognosis (del (11q)), translocations (t (14; 19)), or even gains of entire chromosomes (trisomy 12) [7, 8].

Human leukocyte antigens (HLAs) are transmembrane molecules encoded by the major histocompatibility complex (MHC) that play an important role in the immune system of the human body [9, 10]. Generally, HLA class I proteins bind endogenous peptides and present them to CD8^+^ T cells, leading, in case of activation of the lymphocytes, to cytotoxic destruction of the cell. HLA class II proteins are expressed only on antigen-presenting cells (APCs) and B lymphocytes and present exogenous peptides to CD4^+^ T cells [4, 10–12].

Because HLA genes are extremely polymorphic and virtually ubiquitous, they have been the focus of intense research efforts in the past few years. Many studies have searched for links between HLA genes and different diseases and have established strong links between certain HLA alleles and genotypes and different diseases [13, 14]. One of the first associations was for ankylopoietic spondylitis; it was found that the HLA-B^*∗*^27 alleles were associated with an early onset of the disease and were involved in the occurrence of severe joint manifestations [15]. Other studies focused on rheumatoid arthritis, showing that the association with HLA-DRB1^*∗*^04:01 increases the risk of the disease and the severity of symptoms [16, 17]. A solid body of research also demonstrates the connection between HLA expression and malignant diseases such as Hodgkin's and non-Hodgkin's lymphomas, each with its predisposing alleles, generally HLA class I alleles in the case of Hodgkin's lymphoma (HLA-B^*∗*^05/08/37) [18] and HLA class II alleles for non-Hodgkin's lymphomas (HLA-DRB1^*∗*^03:01/DRB1^*∗*^01:01/DQB1^*∗*^05:01/DPB1^*∗*^03:01) [19, 20].

The involvement of genetic factors in the occurrence of CLL has been investigated using genome-wide association studies (GWAS) [21–24]. Among the genetic factors, some of the genes most strongly associated with the disease are certain allelic variants of HLA genes [25]. However, this association is strongly influenced by ethnic variability; hence, for various populations, different HLA genes are involved in the occurrence of the disease [4]. Several examples for HLA expression associated with different populations include HLA-DRB1^*∗*^04:02 statistically associated with CLL in Jewish populations [4], DRB1^*∗*^04:01 and DRB1^*∗*^07:01 expressed in Iranian patients with CLL [5], or DQB1^*∗*^03:02 and DRB4^*∗*^01:03 correlated with an increased risk of developing the disease in German patients [26]. At the same time, while in Eastern European populations the incidence of CLL has doubled over the past 30 years, there are no studies on HLA expression in patients from this geographical area.

Although HLA typing was serologic in the beginning, it has improved and evolved toward sequence-specific oligonucleotide typing and sequence-specific primer typing [27]. Despite the efficiency of these methods, they have been surpassed by sequence-based typing (SBT), which ensures high-resolution analysis of HLA genes with precise identification of HLA alleles [28]. The data provided by SBT sequencing are comprehensive, and this method represents the gold standard in HLA genotyping. Nevertheless, a new sequencing method named next-generation sequencing (NGS) has been developed [29]. It produces massive and detailed sequencing data, offering a broad view of the genes in their entirety [30]. In addition, it offers a perspective on some insufficiently analyzed HLA class II genes such as HLA-DPA1/DQA1/DRBo (HLA-DRB3/4/5). Owing to its higher sensitivity in detecting low-frequency variants and lower limit of detection, NGS genotyping has become the front-runner tool in the analysis of HLA gene polymorphisms [31].

The aim of this study was to assess, for the first time, the possible associations between HLA genes and CLL in Romanian patients, using NGS genotyping.

## 2. Materials and Methods

### 2.1. Patients and Controls

This study was conducted at Fundeni Clinical Institute, Hematology Clinic, affiliated with “Carol Davila” University of Medicine and Pharmacy, and included patients diagnosed with CLL between 2020 and 2022. In accordance with the Declaration of Helsinki, written consent was obtained from both the patient and control groups. This study was reviewed and approved by the Ethical Committee of Fundeni Clinical Institute (nr. 46893). For consistency, medical data from each patient's medical file were extracted, processed, and analyzed statistically by the academic group involved in the study. Patients with incomplete medical history were excluded from the study. Initially, 113 patients were diagnosed with CLL, but following several inclusion and exclusion criteria, only 66 were included in the study. The control group comprised 100 blood donor volunteers included in the National Registry of Hematopoietic Stem Cell Donors. To avoid bias, the controls included in the study were not related to the patients in the study group; the main sex and age variables can be found in Table 1. The CLL group included patients between 41 and 89 years old with a median age of 63.2 years. Among them, 28 were female (42.4%) and 38 were male (57.5%). All patients were previously diagnosed in compliance with European guidelines for CLL [32, 33]. Several of the most important parameters considered for diagnostic included blood cell counts, peripheral blood smear with evidence of cell morphology, and immunophenotypic analysis. Disease severity was determined using RAI staging [34]. After the patients were confirmed with CLL, they were considered as potential study participants based on the following eligibility criteria: age 18 years or older, able to provide informed consent, pregnancy, active infections, central nervous system disorders, cardiovascular diseases, severe lung diseases, severe kidney diseases, severe allergies, severe autoimmune diseases, lack of other associated cancers or diseases that have a prognosis of less than 5 years, mental disorders that can interfere with the participation in the study, positive serology for hepatitis B and C, and the absence of other chromosomal disorders associated with CLL such as (del (13q), trisomy 12, del (11q), del (17p), and del (6q)) [35]. For each blood donor volunteer participating in the study, medical history was analyzed from the medical personal file, while biochemical parameters and the viral status were assessed, as a national requirement, following the blood donating procedure.

### 2.2. Sample Collection and DNA Extraction

For each patient and control, 5 mL of whole blood was collected, mixed with EDTA, and used for DNA extraction. In the case of leukopenic patients, DNA extraction was postponed until leukocyte numbers increased above 1000/mmc and a new blood sample was collected. If the DNA concentration was less than 20 ng/*µ*L after extraction, a new blood sample was collected and processed.

For DNA extraction, 200 *µ*L of whole blood was used along with a QIAmp DNA Blood Mini® kit (QIAGEN, Hilden, Germany) to obtain a 200 *µ*L eluate. DNA was extracted using a silica membrane. Before extraction, each sample was vortexed well, and 200 *µ*L whole blood was collected and mixed with protease and lysis buffer. The samples were then placed in a thermoblock to facilitate rapid lysis. Once the cell membranes were destroyed and DNA was free, alcohol was added to induce DNA precipitation. The samples were then transferred to special tubes with silica membranes. Upon centrifugation, DNA attaches to the membrane owing to the difference in the electric charge between the two. After washing and purifying DNA, it was separated from the silica membrane by adding an elution buffer to neutralize the electric charges. DNA was collected in separate tubes and stored at −18°C until use. An IMPLEN nanophotometer was used to determine the DNA concentration and purity. Acceptable solutions were those with an A_260nm_/A_280nm_ ratio between 1.7 and 1.9, which certifies the purity of the solution and a DNA concentration >20 ng/*µ*L.

### 2.3. Genotyping Using NGS

For HLA gene polymorphism in patients and controls, HLA genotyping was performed with NGS using Immucor reagents. The MIA FORA NGS MFlex HLA protocol (MIA FORA™ NGS MFlex) was used to type both class I and II HLA genes; a total of 11 loci with all their genes were analyzed. This method involves three important sequences: long-range PCR, library construction, and sequencing and data analysis. In the first step, i.e., long-range PCR, the most relevant class I and II HLA genes were amplified (HLA-A/B/C, HLA-DRB1/3/4/5, and HLA-DQB1/DPB1/DPA1/DQA1). Library construction begins with fragmentation of the samples, followed by end repair and the addition of an adenine nucleotide at the end of each fragment for better ligation of unique index adaptors. Each fragment is barcoded so that it can be easily identified during sequencing. DNA fragments that ranged between 500 and 900 base pairs were selected using the Pippin Prep system. Later, a final amplification of the size-selected library was needed to ensure proper cluster generation. Before preparing the final library, the concentration was assessed using a Qubit® fluorometer (Thermo Fisher Scientific) and adjusted in conformity with the protocol [36].

After the NGS sequencing library was prepared using Illumina reagents, it was loaded onto an Illumina MiniSeq sequencer (Illumina, San Diego, CA, USA). After sequencing was completed, the data were interpreted using the MIA FORA NGS FLEX software (Sirona Genomics, Inc.) and two reference databases: the IMGT and Sirona Genomics databases [36].

### 2.4. Statistical Analysis

To compare allele frequencies between the two groups, statistical analysis was performed using the chi-square test or Fisher's exact test with Yates's correction. Odds ratios (ORs) with 95% confidence intervals (CIs) were calculated to determine the strength of associations. Statistical significance was set at *p*  <  0.05. SPSS V 28.0 was used for the statistical analysis of the results.

## 3. Results

We compared HLA genes in CLL patients with those in the control group. For each CLL patient, we considered both haplotypes, which led to the analysis of 132 alleles for each gene in the patient group. The same rule was applied to the 100 volunteers representing the control group, resulting in 200 alleles as the comparison batch. The exception to this rule for both populations was the HLA-DRB3/4/5 genes, which showed a significantly lower expression in comparison with the rest of the genes and had a lower count. Genotyping of HLA class I (HLA-A/B/C) and class II (HLA-DRB1/3/4/5 and HLA-DQB1/DPB1/DPA1/DQA1) genes was performed using NGS to establish possible connections with CLL. Detailed typing results were collected for both groups. In total, we identified 25 HLA-A alleles, 45 HLA-B alleles, 29 HLA-C alleles, 9 HLA-DPA1 alleles, 20 HLA-DPB1 alleles, 18 HLA-DQA1 alleles, 19 HLA-DQB1 alleles, 31 HLA-DRB1 alleles, 4 HLA-DRB3 alleles, 2 HLA-DRB4 alleles, and 3 HLA-DRB5 alleles.

We evaluated allele frequencies at the 6-digit level and identified 4 protective and 2 predisposing alleles for CLL from both HLA classes (Table 2). Although HLA class I genes are represented less, we identified one allele with a strong protective role: HLA-A^*∗*^24:02:01 (OR = 0.38, *p*=0.01, Supp Table S1).

One of the less studied genes, HLA-DQA1, with its allelic variant HLA-DQA1^*∗*^05:05:01 (*p*=0.01, OR = 0.56, Table 2 & Supp Table S1), was also identified for its protective role, along with HLA-DQB1^*∗*^03:02:01 (*p*=0.03, OR = 0.4, Table 2 and Supp Table S1).

The HLA-DRBo genes (HLA-DRB3/4/5) were distributed differently between the study groups. HLA-DRB4^*∗*^01:03:01 (*p*=0.03, OR = 0.54) was one of the least expressed alleles in the patient group but was well expressed in controls, as shown in Supp Table S1.

**Figure 1:**
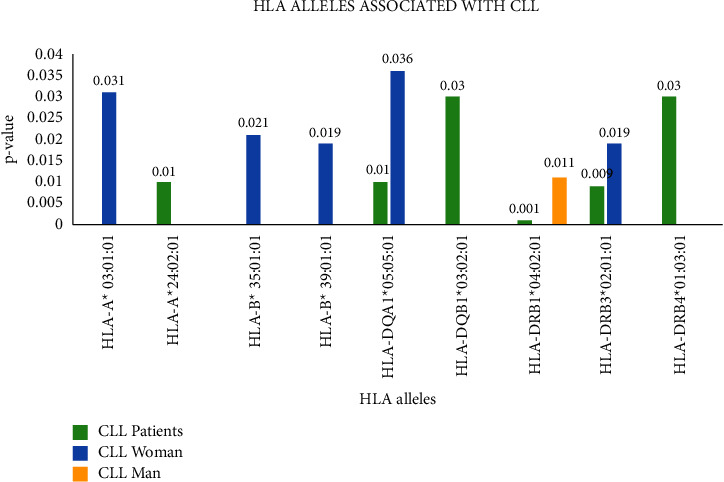
^*∗*^Significant HLA allelic associations for CLL patients. Significant HLA alleles for the CLL group of patients are represented with green, for women with CLL are represented in blue and for men with CLL are represented with yellow.

A key finding of our study was the importance of HLA-DRB1 genes in the development of CLL. In this regard, we identified a strong high-resolution allelic association between HLA-DRB1^*∗*^04:02:01 and CLL (*p*=0.009, OR = 1.03), as shown in Figure 1. Another statistically significant allele from this group was HLA-DRB3^*∗*^02:01:01 (*p*=0.009, Table 2 and Supp Tables S1–S4).

We also evaluated the frequency of HLA alleles with a statistically proven impact on CLL for each of the genders (Table 3). Of the 66 patients included in the study, 28 were women, leading to a number of 56 alleles to be analyzed. For comparison, we selected women from the control group, who were 45 in number, resulting in 90 alleles for testing. We did the same for the 38 male patients (meaning 76 alleles) enrolled in the study, which were compared with 55 healthy men from the control group (meaning 110 alleles).

For the female population with CLL, we identified two alleles associated with the disease HLA-B^*∗*^ 39:01:01 (*p*=0.019, OR = 1.077) and HLA-DRB3^*∗*^ 02:01:01(*p*=0.019, OR = 1.077) together with three alleles with protective role HLA-A^*∗*^ 03:01:01 (*p*=0.031, OR = 0.365), HLA-B^*∗*^ 35:01:01 (*p*=0.021, OR = 0.228), and HLA-DQA1^*∗*^ 05:05:01(*p*=0.036, OR = 0.487) as can be seen in Table 3 and Supp Tables S5–S8.

In the male group of patients, only one statistically significant association was established with HLA-DRB1^*∗*^ 04:02:01 (*p*=0.011, OR = 1.070) which coincides with the predisposing role of this allele for the whole group of CLL patients (see Table 3 and Supp Tables S9–S12).

## 4. Discussion

The following study focused on analyzing a sample of Romanian patients with CLL for HLA expression. The study is the first of its kind for this particular population. Using next-generation sequencing as a method of analysis, we found several alleles that can potentially play protective roles in CLL initiation HLA-A^*∗*^24:02:01 and HLA-DQA1^*∗*^05:05:01 as well as HLA alleles associated with an increased risk for CLL development HLA-DRB1^*∗*^04:02:01 and HLA-DRB3^*∗*^02:01:01. However, given the relatively small number of patients included in the study, the present results need to be validated and expanded on larger cohorts.

Over time, numerous associations between HLA genes and CLL have been established for different ethnic populations [4, 22, 26, 37]. However, to the best of our knowledge, there are no reports on the Romanian population.

Fellow Romanian researchers have established various connections between HLA and certain diseases, such as type 1 diabetes mellitus (T1DM), celiac disease (CD), and chronic renal insufficiency (CRI). For example, one of the first studies conducted by Guja et al. [38] established a strong association between HLA-DQB1 and T1DM. The associations found are both protective and predisposing. Nevertheless, they identified a higher prevalence of protective alleles, which could explain the low incidence of this disease in the Romanian population. Recently, our team identified certain HLA class I and class II alleles with predisposing effects in patients with CRI [39]. Alleles such as HLA-B^*∗*^40, HLA-C^*∗*^12, and HLA-DRB1^*∗*^14 were found to be particularly predisposing to CRI in the Romanian population, in contrast to previous findings. Another important finding was the implication of HLA-DQA1^*∗*^05:01, HLA-DQB1^*∗*^02:01, and HLA-DQB1^*∗*^02:02 in the occurrence of CD in Romanian patients, a connection established by Maruntelu et al. in a 2022 study [40].

These studies inspired us to look further for other possible associations in the Romanian population. In contrast to the work of our contemporaries, which involved low-resolution genotyping, we wanted to use a more potent genotyping method; that is why our data were obtained using NGS genotyping, which offered an extensive picture of patients with HLA class I and II genes. The main advantage of this method is that it allows the collection of additional information about the analyzed alleles, such as synonymous variant data. Another advantage is that it allows the analysis of some less investigated HLA class II genes, such as HLA-DPA1, HLA-DQA1, HLA-DRB3, HLA-DRB4, and HLA-DRB5, which allowed us to establish new associations between CLL and the genes in this class that were predisposing or protective.

In this study, we evaluated 66 CLL patients for possible polymorphisms in 11 HLA genes (HLA-A/B/C, HLA-DRB1/3/4/5, and HLA-DQB1/DPB1/DPA1/DQA1). We compared our results with other previous findings and demonstrated that while the expression of several alleles is consistent with other studies, we have also identified several new statistically significant associations between these genes and CLL.

CLL is characterized by an increase in the number of B lymphocytes with a modified phenotype [41]. This shift of B cells is attributed especially to the genetic factor, with some of the most important genes implicated in this modified response being the HLA genes [42]. These genes encode MHC molecules and thereby influence the immune response, which can thus be stimulated or inhibited [21]. Moreover, modified B lymphocytes are expected to exhibit representative changes in the level of MHC class II genes present specifically on APCs, of which B lymphocytes are also a part. This change is in accordance with our results, which indicate the involvement of HLA-DRB1^*∗*^04:02:01 in the occurrence of CLL. As mentioned earlier, one of the most relevant results of our study shows the association of HLA-DRB1^*∗*^04:02:01 with CLL with a *p* value of 0.001 and OR of 1.05, which demonstrates a strong HLA-disease association. This allele was also found to be associated with the disease in men but not in women participating in the study (*p*=0.011, OR = 1.070). Gragert et al. [4] also reported that HLA-DRB1^*∗*^04:02 was strongly associated with systemic CLL in the American and Jewish populations. This allele was also documented by Scally et al. [43] in the occurrence of another pathology, rheumatoid arthritis, where this time it is mentioned as having a strong protective role.

In addition to this important finding, we have established another positive association between HLA-DRB3^*∗*^02:01:01 and CLL (*p*=0.009, OR = 1.03). The association is also valid for the women group (*p*=0.019, OR = 1.077). Several reports focus on HLA-DRB3 genes and their involvement and association with different pathologies, although their exact role has yet to be established. For example, Mueller et al. [44] found a high expression of the HLA-DRB3^*∗*^01:01 allele among women with CLL. Moreover, Le et al. [45] talked about the involvement of HLA-DRB3^*∗*^02:02 in the occurrence of PLA2R-related membranous nephropathy.

The results of HLA-CLL association studies using classical genotyping are not particularly consistent with our findings. The first protective HLA allele we encountered was HLA-A^*∗*^24:02:01. This finding contradicts the results of a study by Cuttner et al. [46], which indicates that the HLA-A24 alleles are positively associated with the disease in the Ashkenazi Jews population; however, this hypothesis was not statistically supported. The same situation was recorded in a later study that mentioned the HLA-A^*∗*^24 alleles and their higher incidence among CLL patients; however, the association could not be supported after statistical analysis [25]. Several studies have identified HLA-A^*∗*^01:01 as a strong protective allele against the disease [4, 37]. Another strong association between the HLA-A^*∗*^02:01 allele and CLL has been widely reported [4, 47].

Gragert et al. [4] correlated HLA-DQB1^*∗*^03:02:01 with an increased risk of developing CLL. A similar association has been reported for German patients with CLL [26]. Other reports indicate a high risk of progression of high-count monoclonal B lymphocytosis to CLL in patients with IGHV mutations and HLA-DQB1^*∗*^03 [25]. Nevertheless, our study showed a high frequency of HLA-DQB1^*∗*^03:02:01 in the control group; based on this observation, we have an assigned a protective role to it. These findings are supported by the protective role of the same allele in acute lymphoblastic leukemia [48].

The same study that established the connection between HLA-DQB1^*∗*^03:02 and CLL pointed out that the presence of HLA-DRB4∗01:03 was associated with a higher risk of developing CLL [26]. These findings were reinforced by a subsequent study that linked HLA-DRB4^*∗*^01:03 with the disease regardless of the age and sex of the patients [44]. This allele was positively associated with diabetes in a study by Zhao et al. [49]. Although our results contradict previous findings, we determined a strong protective association of the HLA-DRB4^*∗*^01:03:01 allele with CLL. In our study, we also show that the association of this allele is doubled by the association of the HLA-DQB1^*∗*^03:02:01 allele with CLL in a manner similar to that in previous studies.

HLA-DQA1^*∗*^05:05:01 is another allele with a protective role that showed a statistically significant association with the disease. Interestingly enough, this association is further shown to have a protective role for the female group included in the study. Several other reports have identified this gene as being associated with different pathologies, but not in a protective manner as our study suggests. Wang et al. [50] associated the presence of HLA- DQA1^*∗*^05:05 with a high risk of cutaneous squamous cell carcinoma. Schwarm et al. also identified HLA-DQA1^*∗*^05:05 as being strongly associated with the development of bullous pemphigoid in Germans [51]. At the same time, Nowak et al. [52] noted a positive association between HLA-DQA1^*∗*^05 and extensive ulcerative colitis in children.

In addition to the alleles with an impact on the occurrence of CLL, our study also identified a series of alleles that are associated with the disease but without statistical significance, such as: HLA-A^*∗*^03:02:01, HLA-DPA1^*∗*^02:02:02, HLA-DQA1^*∗*^01:02:01, and HLA-DRB1^*∗*^11:01:01. An important discovery was HLA-B^*∗*^35:01:01 allele that approaches the limit of statistical significance with *p*=0.057 and has a proven protective role for women. In a previous study, the HLA-B35 antigen was found to be more prevalent in Ashkenazi Jews of European origin as well as in Caucasian patients with CLL [46]. In our case, we encountered a strong predisposing role of HLA-B∗39:01:01 in the occurrence of the disease in females. Similar results were obtained by Hojjat-Farsangi et al. [37], who reported a higher incidence of HLA-B^*∗*^35:01 in CLL patients, in addition to two HLA-B alleles with a protective role (HLA-B53:01 and HLA-B^*∗*^65:01).

Although our study did not highlight the HLA-DRB1^*∗*^11:01:01 allele as having statistical significance, it approaches the limit of statistical significance with a *p* value of 0.057. DiBernardo et al. [47] found a link between HLA-DRB1^*∗*^11:01 and CLL, albeit without statistical relevance. The involvement in these alleles in the occurrence of various cancers has been documented by Arons et al. [53], who reported that HLA-DRB1^*∗*^11 is a potential risk factor for hairy cell leukemia. Aureli et al. [54] noticed an association between this allele and different types of cancer, i.e., breast cancer genetically linked with HLA-DRB1^*∗*^11:01. Moreover, Rossman et al. [55] reported the importance of HLA-DRB1^*∗*^11:01 as a susceptibility factor for sarcoidosis. Interestingly, this allele also seems to have a protective role in juvenile idiopathic arthritis, as mentioned by Rezaieyazdi et al. [56].

Other studies have also identified strong links between CLL and HLA genes such as DRB1^*∗*^04:01 [4, 5, 26, 44], DRB1^*∗*^07:01 [4, 5], DQB1^*∗*^03:02 [4, 26, 44], and DRB4^*∗*^01:03 [26, 44]. The diversity offered by the results of these studies can be attributed to variations in the genetic background; this should be considered in future studies.

While our data report novel and interesting correlations between CLL and specific HLA alleles, several limitations have to be taken into consideration and current results need further validation. One major limitation of the present study is the small number of patients included, due to the relative rare presence of CLL and to the inclusion/exclusion criteria employed. Another issue that needs to the addressed in future studies involves a better statistical match between controls and patients for both gender and age variables.

## 5. Conclusions

Our preliminary results show that HLA-DRB1^*∗*^04:02:01 and HLA-DRB3^*∗*^02:01:01 alleles correlate with CLL in Romanian patients. HLA-A^*∗*^24:02:01 and HLA-DQA1^*∗*^05:05:01 could be associated with a lower risk of CLL development. Moreover, HLA-B^*∗*^39:01:01 and HLA-DRB3^*∗*^02:01:01 are predisposing for women, while HLA-DRB1^*∗*^04:02:01 increases the risk of CLL for men.

## Figures and Tables

**Table 1:** Cases and control general characteristics.

	Total	Gender		Age (years)
		Male (%)	Female (%)	Mean
CLL patients	66	57.5	42.4	63.2
Controls	100	55	45	34.3

CLL: chronic lymphocytic leukemia.

**Table 2:** Distribution of HLA alleles in CLL patients and the control group.

Allele	Cases *n*1		Controls *n*2		*P* value	OR	95% CI	
	Number	(%)	Number	(%)			Low	Upper
HLA-A^*∗*^24:02:01	17	12.9	10	5.0	0.01	0.38	0.183	0.822
HLA-DQA1^*∗*^05:05:01	29	22.0	24	12.0	0.01	0.56	0.333	0.895
HLA-DQB1^*∗*^03:02:01	13	9.8	8	4.0	0.03	0.40	0.173	0.953
HLA-DRB1^*∗*^04:02:01	7	5.3	0	0.0	0.001	1.05	1.014	1.100
HLA-DRB3^*∗*^02:01:01	5	3.8	0	0.0	0.009	1.03	1.005	1.075
HLA-DRB4^*∗*^01:03:01	23	17.4	19	9.5	0.03	0.54	0.309	0.961

^*∗*^Statistical significance was determined after calculating the *p* value, OR (odds ratio), and CI (confidence interval). The chi-square test or Fisher's test was used to estimate the differences between the CLL patient and control groups; *n*: number of alleles in the patient and control groups. A complete listing of alleles associated with the disease is provided in Supplemental Tables S1–S4. Comparison of the most important HLA alleles at the 6-digit level between CLL patients and the control group.

**Table 3:** Distribution of HLA alleles for males and females in CLL patients and the control group.

Allele	Gender	Cases		Controls		*P* value	OR	95% CI	
		Number	(%)	Number	(%)			Low	Upper
HLA-A^*∗*^03:01:01	Female	10	17.8	6	6.5	0.031	0.365	0.140	0.950
HLA-B^*∗*^35:01:01	Female	8	14.2	3	3.2	0.021	0.228	0.063	0.825
HLA-B^*∗*^39:01:01	Female	4	7.14	0	0	0.019	1.077	1.001	1.158
HLA-DQA1^*∗*^05:05:01	Female	15	26.7	12	13.04	0.036	0.487	0.246	0.964
HLA-DRB3^*∗*^02:01:01	Female	4	7.14	0	0	0.019	1.077	1.001	1.158
HLA-DRB1^*∗*^04:02:01	Male	5	6.5	0	0	0.011	1.070	1.008	1.136

^*∗*^Statistical significance was determined after calculating the *p* value, OR (odds ratio), and CI (confidence interval). The chi-square test or Fisher's test was used to estimate the differences between the CLL patient and control groups; *n*: number of alleles in the patient and control groups. A complete listing of alleles associated with the disease for each gender is provided in Supplemental Tables S5–S12. Comparison of most important HLA alleles at the 6-digit level between CLL patients and the control group for both genders.

## Data Availability

The data that support the findings of this study are available on request.

## Supplementary Materials

Supplementary Materials The following supporting information can be downloaded from Supp Table S1: Supplemental Table 1: distribution of HLA alleles in CLL patients and the control group. Comparison of most important HLA alleles at the 6-digit levels between CLL patients and the control group. Supp Table S2: Supplemental Table 2: distribution of HLA-DRB3 in CLL patients and the control group. Comparison of most important HLA alleles at the 6-digit levels between CLL patients and the control group. Supp Table S3: Supplemental Table 3: distribution of HLA-DRB4 in CLL patients and the control group. Comparison of most important HLA alleles at the 6-digit levels between CLL patients and the control group. Supp Table S4: Supplemental Table 4: distribution of HLA-DRB5 in CLL patients and the control group. Comparison of most important HLA alleles at the 6-digit levels between CLL patients and the control group. Supp Table S5: Supplemental Table 5: distribution of HLA alleles in CLL female patients and the female control group. Comparison of most important HLA alleles at the 6-digit levels between CLL women and the women in the control group. Supp Table S6: Supplemental Table 6: distribution of HLA-DRB3 in CLL women patients and the women control group. Comparison of most important HLA alleles at the 6-digit levels between CLL women and the women in the control group. Supp Table S7: Supplemental Table 7: distribution of HLA-DRB4 in CLL women patients and the women control group. Comparison of most important HLA alleles at the 6-digit levels between CLL women and the women in the control group. Supp Table S8: Supplemental Table 8: distribution of HLA-DRB5 in CLL women patients and the women control group. Comparison of most important HLA alleles at the 6-digit levels between CLL women and the women in the control group. Supp Table S9: Supplemental Table 9: distribution of HLA alleles in CLL male patients and the male control group. Comparison of most important HLA alleles at the 6-digit levels between CLL men and the men in the control group. Supp Table S10: Supplemental Table 10: distribution of HLA-DRB3 in CLL men patients and the men control group. Comparison of most important HLA alleles at the 6-digit levels between CLL men and the men in the control group. Supp Table S11: Supplemental Table 11: distribution of HLA-DRB4 in CLL men patients and the men control group. Comparison of most important HLA alleles at the 6-digit levels between CLL men and the men in the control group. Supp Table S12: Supplemental Table 12: distribution of HLA-DRB5 in CLL men patients and the men control group. Comparison of most important HLA alleles at the 6-digit levels between CLL men and the men in the control group.
