# Supplementary material for: HLA Gene Polymorphisms in Romanian Patients with Chronic Lymphocytic Leukemia
Source: Genet Res (Camb). 2024 Feb 28;2024:8852876. doi: 10.1155/2024/8852876 (PMC10917483; doi:10.1155/2024/8852876)
Supplement: Supplementary Materials — The following supporting information can be downloaded from Supp Table S1: Supplemental Table 1: distribution of HLA alleles in CLL patients and the control group. Comparison of most important HLA alleles at the 6-digit levels between CLL patients and the control group. Supp Table S2: Supplemental Table 2: distribution of HLA-DRB3 in CLL patients and the control group. Comparison of most important HLA alleles at the 6-digit levels between CLL patients and the control group. Supp Table S3: Supplemental Table 3: distribution of HLA-DRB4 in CLL patients and the control group. Comparison of most important HLA alleles at the 6-digit levels between CLL patients and the control group. Supp Table S4: Supplemental Table 4: distribution of HLA-DRB5 in CLL patients and the control group. Comparison of most important HLA alleles at the 6-digit levels between CLL patients and the control group. Supp Table S5: Supplemental Table 5: distribution of HLA alleles in CLL female patients and the female control group. Comparison of most important HLA alleles at the 6-digit levels between CLL women and the women in the control group. Supp Table S6: Supplemental Table 6: distribution of HLA-DRB3 in CLL women patients and the women control group. Comparison of most important HLA alleles at the 6-digit levels between CLL women and the women in the control group. Supp Table S7: Supplemental Table 7: distribution of HLA-DRB4 in CLL women patients and the women control group. Comparison of most important HLA alleles at the 6-digit levels between CLL women and the women in the control group. Supp Table S8: Supplemental Table 8: distribution of HLA-DRB5 in CLL women patients and the women control group. Comparison of most important HLA alleles at the 6-digit levels between CLL women and the women in the control group. Supp Table S9: Supplemental Table 9: distribution of HLA alleles in CLL male patients and the male control group. Comparison of most important HLA alleles at the 6-digit levels bet [file 8852876.f1.zip › Supplemental Table 1 (2).docx]

**Supplemental Table 1.** Distribution of HLA alleles in CLL-patients and the control group. Comparison of most important HLA alleles at the 6-digit levels between CLL patients and the control group.

| Allele | Cases  *n1* = 132 | Controls  *n2* = 200 | *p*-value | OR | 95% CI | |
| --- | --- | --- | --- | --- | --- | --- |
|  | Number | Number |  |  | Low | Upper |
| HLA-A 01:01:01 | 14 | 28 | 0.36 | 1.32 | 0.722 | 2.412 |
| HLA-A 02:01:01 | 38 | 59 | 0.88 | 1.02 | 0.727 | 1.445 |
| HLA-A 02:02:01 | 0 | 2 | 0.52 | 0.99 | 0.976 | 1.004 |
| HLA-A 02:05:01 | 3 | 1 | 0.3 | 0.22 | 0.023 | 2.092 |
| HLA-A 03:01:01 | 18 | 20 | 0.3 | 0.73 | 0.403 | 1.333 |
| HLA-A 03:02:01 | 3 | 0 | 0.06 | 1.02 | 0.997 | 1.050 |
| HLA-A 11:01:01 | 4 | 8 | 0.76 | 1.32 | 0.406 | 4.295 |
| HLA-A 24:02:01 | 17 | 10 | 0.01 | 0.38 | 0.183 | 0.822 |
| HLA-A 24:03:01 | 2 | 0 | 0.15 | 1.01 | 0.994 | 1.037 |
| HLA-A 25:01:01 | 6 | 5 | 0.35 | 0.55 | 0.171 | 1.1766 |
| HLA-A 26:01:01 | 5 | 8 | 0.92 | 1.05 | 0.353 | 3.158 |
| HLA-A 29:01:01 | 1 | 1 | 1.00 | 0.66 | 0.42 | 10.460 |
| HLA-A 29:02:01 | 1 | 4 | 0.65 | 2.64 | 0.298 | 23.360 |
| HLA-A 30:01:01 | 1 | 8 | 0.09 | 5.28 | 0.668 | 41.726 |
| HLA-A 30:04:01 | 0 | 3 | 0.27 | 0.98 | 0.968 | 1.002 |
| HLA-A 31:01:02 | 0 | 3 | 0.27 | 0.98 | 0.968 | 1.002 |
| HLA-A 32:01:01 | 7 | 14 | 0.53 | 1.32 | 0.547 | 3.183 |
| HLA-A 33:01:01 | 1 | 1 | 1.00 | 0.66 | 0.042 | 10.460 |
| HLA-A 33:01:02 | 1 | 1 | 1.00 | 0.66 | 0.042 | 10.460 |
| HLA-A 33:03:01 | 0 | 3 | 0.27 | 0.98 | 0.968 | 1.002 |
| HLA-A 66:01:01 | 1 | 0 | 0.39 | 1.00 | 0.993 | 1.023 |
| HLA-A 66:02:01 | 0 | 1 | 1.00 | 0.99 | 0.985 | 1.005 |
| HLA-A 68:01:01 | 1 | 3 | 1.00 | 1.98 | 0.208 | 18.832 |
| HLA-A 68:01:02 | 1 | 8 | 0.09 | 5.28 | 0.668 | 41.726 |
| HLA-A 68:02:01 | 2 | 0 | 0.15 | 1.01 | 0.994 | 1.037 |
| HLA-B 07:01:01 | 1 | 0 | 0.39 | 1.00 | 0.993 | 1.023 |
| HLA-B 07:02:01 | 6 | 9 | 0.98 | 0.99 | 0.361 | 2.716 |
| HLA-B 07:05:01 | 1 | 0 | 0.39 | 1.00 | 0.993 | 1.023 |
| HLA-B 08:01:01 | 9 | 19 | 0.38 | 1.39 | 0.650 | 2.985 |
| HLA-B 13:02:01 | 4 | 10 | 0.38 | 1.65 | 0.528 | 5.151 |
| HLA-B 14:01:01 | 0 | 1 | 1.00 | 0.99 | 0.985 | 1.005 |
| HLA-B 14:02:01 | 2 | 3 | 1.00 | 0.99 | 0.168 | 5.845 |
| HLA-B 15:01:01 | 2 | 2 | 0.65 | 0.66 | 0.094 | 4.628 |
| HLA-B 15:03:01 | 0 | 1 | 1.00 | 0.99 | 0.985 | 1.005 |
| HLA-B 15:10:01 | 0 | 1 | 1.00 | 0.99 | 0.985 | 1.005 |
| HLA-B 15:24:01 | 1 | 0 | 0.39 | 1.00 | 0.993 | 1.023 |
| HLA-B 18:01:01 | 15 | 17 | 0.38 | 0.74 | 0.387 | 1.445 |
| HLA-B 18:03:01 | 0 | 1 | 1.00 | 0.99 | 0.985 | 1.005 |
| HLA-B 18:04:01 | 0 | 1 | 1.00 | 0.99 | 0.985 | 1.005 |
| HLA-B 18:05:01 | 5 | 2 | 0.11 | 0.26 | 0.052 | 1.341 |
| HLA-B 27:02:01 | 3 | 5 | 1.00 | 1.10 | 0.267 | 4.525 |
| HLA-B 27:05:02 | 3 | 4 | 1.00 | 0.88 | 0.200 | 3.869 |
| HLA-B 35:01:01 | 10 | 6 | 0.05 | 0.39 | 0.147 | 1.064 |
| HLA-B 35:02:01 | 2 | 7 | 0.32 | 2.31 | 0.487 | 10.949 |
| HLA-B 35:03:01 | 7 | 6 | 0.29 | 0.56 | 0.194 | 1.646 |
| HLA-B 35:08:01 | 0 | 1 | 1.00 | 0.99 | 0.985 | 1.005 |
| HLA-B 37:01:01 | 0 | 3 | 0.27 | 0.98 | 0.968 | 1.002 |
| HLA-B 38:01:01 | 8 | 6 | 0.17 | 0.49 | 0.176 | 1.394 |
| HLA-B 39:01:01 | 5 | 2 | 0.11 | 0.26 | 0.052 | 1.341 |
| HLA-B 39:05:01 | 0 | 1 | 1.00 | 0.99 | 0.985 | 1.005 |
| HLA-B 39:31:01 | 0 | 1 | 1.00 | 0.99 | 0.985 | 1.005 |
| HLA-B 40:01:01 | 0 | 2 | 0.52 | 0.99 | 0.976 | 1.004 |
| HLA-B 40:01:02 | 0 | 2 | 0.52 | 0.99 | 0.976 | 1.004 |
| HLA-B 40:02:01 | 0 | 6 | 0.08 | 0.97 | 0.947 | 0.994 |
| HLA-B 40:06:01 | 0 | 1 | 1.00 | 0.99 | 0.985 | 1.005 |
| HLA-B 41:01:01 | 2 | 5 | 0.7 | 1.65 | 0.325 | 8.380 |
| HLA-B 41:02:01 | 3 | 3 | 0.68 | 0.66 | 0.135 | 3.221 |
| HLA-B 44:02:01 | 6 | 11 | 0.69 | 1.21 | 0.459 | 3.192 |
| HLA-B 44:03:01 | 8 | 8 | 0.39 | 0.66 | 0.254 | 1.715 |
| HLA-B 49:01:01 | 4 | 5 | 0.74 | 0.85 | 0.226 | 3.016 |
| HLA-B 50:01:01 | 3 | 0 | 0.06 | 1.02 | 0.997 | 1.050 |
| HLA-B 51:01:01 | 11 | 23 | 0.35 | 1.38 | 0.696 | 2.735 |
| HLA-B 51:05:01 | 1 | 0 | 0.39 | 1.00 | 0.993 | 1.023 |
| HLA-B 52:01:01 | 4 | 4 | 0.71 | 0.66 | 0.168 | 2.593 |
| HLA-B 55:01:01 | 4 | 5 | 0.74 | 0.82 | 0.226 | 3.016 |
| HLA-B 56:01:01 | 0 | 2 | 0.52 | 0.99 | 0.976 | 1.004 |
| HLA-B 58:01:01 | 1 | 2 | 1.00 | 1.32 | 0.121 | 14.411 |
| HLA-B 58:02:01 | 0 | 1 | 1.00 | 0.99 | 0.985 | 1.005 |
| HLA-B 59:01:01 | 0 | 1 | 1.00 | 0.99 | 0.985 | 1.005 |
| HLA-B 81:01:01 | 0 | 1 | 1.00 | 0.99 | 0.985 | 1.005 |
| HLA-C 01:02:01 | 5 | 11 | 0.47 | 1.45 | 0.516 | 4.084 |
| HLA-C 02:02:02 | 5 | 16 | 0.12 | 2.11 | 0.793 | 5.626 |
| HLA-C 03:02:01 | 1 | 3 | 1.00 | 1.98 | 0.208 | 18.832 |
| HLA-C 03:02:02 | 0 | 1 | 1.00 | 0.99 | 0.985 | 1.005 |
| HLA-C 03:03:01 | 6 | 3 | 0.16 | 0.33 | 0.084 | 1.297 |
| HLA-C 03:04:01 | 1 | 4 | 0.65 | 2.64 | 0.298 | 23.360 |
| HLA-C 03:04:02 | 0 | 1 | 1.00 | 0.99 | 0.985 | 1.005 |
| HLA-C 04:01:01 | 26 | 29 | 0.21 | 0.73 | 0.455 | 1.192 |
| HLA-C 05:01:01 | 2 | 4 | 1.00 | 1.32 | 0.245 | 7.104 |
| HLA-C 06:02:01 | 6 | 15 | 0.27 | 1.65 | 0.657 | 4.144 |
| HLA-C 07:01:01 | 21 | 33 | 0.88 | 1.03 | 0.628 | 1.712 |
| HLA-C 07:02:01 | 10 | 11 | 0.44 | 0.72 | 0.317 | 1.661 |
| HLA-C 07:04:01 | 1 | 3 | 1.00 | 1.98 | 0.208 | 18.832 |
| HLA-C 08:02:01 | 2 | 4 | 1.00 | 1.32 | 0.245 | 7.104 |
| HLA-C 12:02:01 | 3 | 0 | 0.06 | 1.02 | 0.997 | 1.050 |
| HLA-C 12:02:02 | 1 | 6 | 0.25 | 3.96 | 0.482 | 32.518 |
| HLA-C 12:03:01 | 19 | 22 | 0.35 | 0.76 | 0.431 | 1.356 |
| HLA-C 12:12:01 | 0 | 1 | 1.00 | 0.99 | 0.985 | 1.005 |
| HLA-C 14:02:01 | 2 | 3 | 1.00 | 0.99 | 0.168 | 5.845 |
| HLA-C 15:02:01 | 4 | 8 | 0.76 | 1.32 | 0.406 | 4.295 |
| HLA-C 15:04:01 | 1 | 0 | 0.39 | 1.00 | 0.993 | 1.023 |
| HLA-C 15:05:01 | 2 | 0 | 0.15 | 1.01 | 0.994 | 1.037 |
| HLA-C 15:13:01 | 0 | 2 | 0.52 | 0.99 | 0.976 | 1.004 |
| HLA-C 16:01:01 | 2 | 4 | 1.00 | 1.32 | 0.245 | 7.104 |
| HLA-C 16:02:01 | 0 | 2 | 0.52 | 0.99 | 0.976 | 1.004 |
| HLA-C 16:04:01 | 4 | 3 | 0.44 | 0.49 | 0.113 | 2.176 |
| HLA-C 17:01:01 | 2 | 4 | 1.00 | 1.32 | 0.245 | 7.104 |
| HLA-C 17:03:01 | 3 | 4 | 1.00 | 0.88 | 0.200 | 3.869 |
| HLA-C 18:01:01 | 0 | 1 | 1.00 | 0.99 | 0.985 | 1.005 |
| HLA-DPA1 01:03:01 | 108 | 152 | 0.2 | 0.92 | 0.831 | 1.039 |
| HLA-DPA1 01:05:01 | 0 | 1 | 1.00 | 0.99 | 0.985 | 1.005 |
| HLA-DPA1 02:01:01 | 16 | 22 | 0.75 | 0.90 | 0.495 | 1.662 |
| HLA-DPA1 02:01:02 | 5 | 13 | 0.28 | 1.71 | 0.626 | 4.701 |
| HLA-DPA1 02:02:02 | 1 | 10 | 0.05 | 6.60 | 0.855 | 50.953 |
| HLA-DPA1 02:04:01 | 1 | 0 | 0.39 | 1.00 | 0.993 | 1.023 |
| HLA-DPA1 02:07:01 | 0 | 1 | 1.00 | 0.99 | 0.985 | 1.005 |
| HLA-DPA1 03:01:01 | 0 | 1 | 1.00 | 0.99 | 0.985 | 1.005 |
| HLA-DPA1 04:01:01 | 1 | 0 | 0.39 | 1.00 | 0.993 | 1.023 |
| HLA-DPB1 01:01:01 | 5 | 16 | 0.12 | 2.11 | 0.793 | 5.626 |
| HLA-DPB1 02:01:02 | 24 | 31 | 0.52 | 0.85 | 0.525 | 1.385 |
| HLA-DPB1 02:02:01 | 0 | 1 | 1.00 | 0.99 | 0.985 | 1.005 |
| HLA-DPB1 03:01:01 | 11 | 15 | 0.78 | 0.90 | 0.427 | 1.898 |
| HLA-DPB1 04:01:01 | 45 | 69 | 0.93 | 1.01 | 0.746 | 1.372 |
| HLA-DPB1 04:02:01 | 24 | 34 | 0.78 | 0.93 | 0.582 | 1.502 |
| HLA-DPB1 05:01:01 | 2 | 7 | 0.32 | 2.31 | 0.487 | 10.949 |
| HLA-DPB1 06:01:01 | 0 | 1 | 1.00 | 0.99 | 0.985 | 1.005 |
| HLA-DPB1 09:01:01 | 1 | 3 | 1.00 | 1.98 | 0.208 | 18.832 |
| HLA-DPB1 10:01:01 | 4 | 6 | 1.00 | 0.99 | 0.285 | 3.441 |
| HLA-DPB1 11:01:01 | 2 | 0 | 0.15 | 1.01 | 0.994 | 1.037 |
| HLA-DPB1 13:01:01 | 2 | 1 | 0.56 | 0.33 | 0.030 | 3.603 |
| HLA-DPB1 14:01:01 | 2 | 1 | 0.56 | 0.33 | 0.030 | 3.603 |
| HLA-DPB1 15:01:01 | 0 | 1 | 1.00 | 0.99 | 0.985 | 1.005 |
| HLA-DPB1 17:01:01 | 2 | 7 | 0.32 | 2.31 | 0.487 | 10.949 |
| HLA-DPB1 18:01:01 | 0 | 1 | 1.00 | 0.99 | 0.985 | 1.005 |
| HLA-DPB1 23:01:01 | 1 | 1 | 1.00 | 0.66 | 0.042 | 10.460 |
| HLA-DPB1 28:01:01 | 2 | 0 | 0.15 | 1.01 | 0.994 | 1.037 |
| HLA-DPB1 104:01:01 | 1 | 3 | 1.00 | 1.98 | 0.208 | 18.832 |
| HLA-DPB1 105:01:01 | 0 | 1 | 1.00 | 0.99 | 0.985 | 1.005 |
| HLA-DQA1 01:01:01 | 7 | 19 | 0.16 | 1.79 | 0.775 | 4.142 |
| HLA-DQA1 01:02:01 | 15 | 38 | 0.06 | 1.67 | 0.959 | 2.915 |
| HLA-DQA1 01:02:02 | 6 | 18 | 0.12 | 1.98 | 0.807 | 4.857 |
| HLA-DQA1 01:03:01 | 10 | 11 | 0.44 | 0.72 | 0.317 | 1.661 |
| HLA-DQA1 01:04:01 | 5 | 5 | 0.52 | 0.66 | 0.195 | 2.235 |
| HLA-DQA1 01:04:02 | 0 | 1 | 1.00 | 0.99 | 0.985 | 1.005 |
| HLA-DQA1 01:05:01 | 3 | 2 | 0.39 | 0.44 | 0.075 | 2.598 |
| HLA-DQA1 01:05:02 | 0 | 1 | 1.00 | 0.99 | 0.985 | 1.005 |
| HLA-DQA1 02:01:01 | 14 | 20 | 0.85 | 0.98 | 0.494 | 1.800 |
| HLA-DQA1 03:01:01 | 15 | 12 | 0.08 | 0.52 | 0.255 | 1.092 |
| HLA-DQA1 03:02:01 | 1 | 0 | 0.39 | 1.00 | 0.993 | 1.023 |
| HLA-DQA1 03:03:01 | 2 | 1 | 0.56 | 0.33 | 0.030 | 3.603 |
| HLA-DQA1 04:01:01 | 1 | 3 | 1.00 | 1.98 | 0.208 | 18.832 |
| HLA-DQA1 05:01:01 | 22 | 43 | 0.27 | 1.29 | 0.811 | 2.052 |
| HLA-DQA1 05:03:01 | 1 | 0 | 0.39 | 1.00 | 0.993 | 1.023 |
| HLA-DQA1 05:05:01 | 29 | 24 | 0.01 | 0.56 | 0.333 | 0.895 |
| HLA-DQA1 05:09:01 | 0 | 1 | 1.00 | 0.99 | 0.985 | 1.005 |
| HLA-DQA1 06:01:01 | 1 | 1 | 1.00 | 0.66 | 0.042 | 10.460 |
| HLA-DQB1 02:01:01 | 16 | 31 | 0.38 | 1.27 | 0.729 | 2.243 |
| HLA-DQB1 02:02:01 | 10 | 13 | 0.70 | 0.85 | 0.388 | 1.899 |
| HLA-DQB1 03:01:01 | 32 | 44 | 0.63 | 0.90 | 0.609 | 1.352 |
| HLA-DQB1 03:02:01 | 13 | 8 | 0.03 | 0.40 | 0.173 | 0.953 |
| HLA-DQB1 03:03:02 | 1 | 2 | 1.00 | 1.32 | 0.121 | 14.411 |
| HLA-DQB1 03:04:01 | 0 | 1 | 1.00 | 0.99 | 0.985 | 1.005 |
| HLA-DQB1 03:19:01 | 0 | 1 | 1.00 | 0.99 | 0.985 | 1.005 |
| HLA-DQB1 04:02:01 | 1 | 3 | 1.00 | 1.98 | 0.208 | 18.832 |
| HLA-DQB1 05:01:01 | 10 | 19 | 0.54 | 1.25 | 0.602 | 2.611 |
| HLA-DQB1 05:02:01 | 16 | 34 | 0.22 | 1.40 | 0.808 | 2.436 |
| HLA-DQB1 05:03:01 | 7 | 8 | 0.57 | 0.75 | 0.280 | 2.031 |
| HLA-DQB1 05:04:01 | 0 | 1 | 1.00 | 0.99 | 0.985 | 1.005 |
| HLA-DQB1 06:01:01 | 5 | 2 | 0.11 | 0.26 | 0.052 | 1.341 |
| HLA-DQB1 06:02:01 | 10 | 15 | 0.98 | 0.99 | 0.459 | 2.137 |
| HLA-DQB1 06:03:01 | 7 | 10 | 0.9 | 0.94 | 0.368 | 2.415 |
| HLA-DQB1 06:03:11 | 0 | 1 | 1.00 | 0.99 | 0.985 | 1.005 |
| HLA-DQB1 06:04:01 | 2 | 5 | 0.7 | 1.65 | 0.325 | 8.380 |
| HLA-DQB1 06:09:01 | 0 | 2 | 0.52 | 0.99 | 0.976 | 1.004 |
| HLA-DRB1 01:01:01 | 6 | 15 | 0.27 | 1.65 | 0.657 | 4.144 |
| HLA-DRB1 01:02:01 | 0 | 1 | 1.00 | 0.99 | 0.985 | 1.005 |
| HLA-DRB1 03:01:01 | 12 | 25 | 0.33 | 1.37 | 0.716 | 2.640 |
| HLA-DRB1 03:02:01 | 0 | 1 | 1.00 | 0.99 | 0.985 | 1.005 |
| HLA-DRB1 04:01:01 | 2 | 4 | 1.00 | 1.32 | 0.245 | 7.104 |
| HLA-DRB1 04:02:01 | 7 | 0 | 0.001 | 1.05 | 1.014 | 1.100 |
| HLA-DRB1 04:03:01 | 3 | 2 | 0.39 | 0.44 | 0.075 | 2.598 |
| HLA-DRB1 04:04:01 | 0 | 2 | 0.52 | 0.99 | 0.976 | 1.004 |
| HLA-DRB1 04:05:01 | 3 | 1 | 0.3 | 0.22 | 0.023 | 2.092 |
| HLA-DRB1 04:07:01 | 1 | 0 | 0.39 | 1.00 | 0.993 | 1.023 |
| HLA-DRB1 07:01:01 | 12 | 28 | 0.17 | 1.54 | 0.812 | 2.919 |
| HLA-DRB1 08:01:01 | 1 | 2 | 1.00 | 1.32 | 0.121 | 14.411 |
| HLA-DRB1 08:03:02 | 1 | 1 | 1.00 | 0.66 | 0.042 | 10.460 |
| HLA-DRB1 09:01:02 | 1 | 0 | 0.39 | 1.00 | 0.993 | 1.023 |
| HLA-DRB1 10:01:01 | 3 | 3 | 0.68 | 0.66 | 0.135 | 3.221 |
| HLA-DRB1 11:01:01 | 11 | 7 | 0.05 | 0.42 | 0.167 | 1.056 |
| HLA-DRB1 11:02:01 | 0 | 2 | 0.52 | 0.99 | 0.976 | 1.004 |
| HLA-DRB1 11:03:01 | 0 | 3 | 0.27 | 0.98 | 0.968 | 1.002 |
| HLA-DRB1 11:04:01 | 13 | 22 | 0.73 | 1.11 | 0.583 | 2.138 |
| HLA-DRB1 12:01:01 | 2 | 4 | 1.00 | 1.32 | 0.245 | 7.104 |
| HLA-DRB1 13:01:01 | 7 | 11 | 0.93 | 1.03 | 0.413 | 2.607 |
| HLA-DRB1 13:02:01 | 2 | 9 | 0.21 | 2.97 | 0.652 | 13.530 |
| HLA-DRB1 13:03:01 | 5 | 5 | 0.52 | 0.66 | 0.195 | 2.235 |
| HLA-DRB1 13:05:01 | 0 | 1 | 1.00 | 0.99 | 0.985 | 1.005 |
| HLA-DRB1 14:01:01 | 6 | 3 | 0.16 | 0.33 | 0.084 | 1.297 |
| HLA-DRB1 14:04:01 | 1 | 1 | 1.00 | 0.66 | 0.042 | 10.460 |
| HLA-DRB1 14:54:01 | 0 | 5 | 0.16 | 0.97 | 0.954 | 0.997 |
| HLA-DRB1 15:01:01 | 11 | 14 | 0.67 | 0.84 | 0.393 | 1.793 |
| HLA-DRB1 15:02:01 | 6 | 3 | 0.16 | 0.33 | 0.084 | 1.297 |
| HLA-DRB1 16:01:01 | 16 | 20 | 0.54 | 0.82 | 0.444 | 1.533 |
| HLA-DRB1 16:02:01 | 0 | 4 | 0.15 | 0.98 | 0.961 | 1.000 |
|  |  |  |  |  |  |  |

* Statistical significance was determined after calculating the *p*-value, OR, and CI. The chi-square test or Fisher’s test was used to estimate the differences between the CLL patient and control groups; *n*: number of alleles in the patient and control groups.
