# Supplementary material for: HLA Gene Polymorphisms in Romanian Patients with Chronic Lymphocytic Leukemia
Source: Genet Res (Camb). 2024 Feb 28;2024:8852876. doi: 10.1155/2024/8852876 (PMC10917483; doi:10.1155/2024/8852876)
Supplement: Supplementary Materials — The following supporting information can be downloaded from Supp Table S1: Supplemental Table 1: distribution of HLA alleles in CLL patients and the control group. Comparison of most important HLA alleles at the 6-digit levels between CLL patients and the control group. Supp Table S2: Supplemental Table 2: distribution of HLA-DRB3 in CLL patients and the control group. Comparison of most important HLA alleles at the 6-digit levels between CLL patients and the control group. Supp Table S3: Supplemental Table 3: distribution of HLA-DRB4 in CLL patients and the control group. Comparison of most important HLA alleles at the 6-digit levels between CLL patients and the control group. Supp Table S4: Supplemental Table 4: distribution of HLA-DRB5 in CLL patients and the control group. Comparison of most important HLA alleles at the 6-digit levels between CLL patients and the control group. Supp Table S5: Supplemental Table 5: distribution of HLA alleles in CLL female patients and the female control group. Comparison of most important HLA alleles at the 6-digit levels between CLL women and the women in the control group. Supp Table S6: Supplemental Table 6: distribution of HLA-DRB3 in CLL women patients and the women control group. Comparison of most important HLA alleles at the 6-digit levels between CLL women and the women in the control group. Supp Table S7: Supplemental Table 7: distribution of HLA-DRB4 in CLL women patients and the women control group. Comparison of most important HLA alleles at the 6-digit levels between CLL women and the women in the control group. Supp Table S8: Supplemental Table 8: distribution of HLA-DRB5 in CLL women patients and the women control group. Comparison of most important HLA alleles at the 6-digit levels between CLL women and the women in the control group. Supp Table S9: Supplemental Table 9: distribution of HLA alleles in CLL male patients and the male control group. Comparison of most important HLA alleles at the 6-digit levels bet [file 8852876.f1.zip › Supplemental Table 4 (2).docx]

**Supplemental Table 4.** Distribution of HLA-DRB5 in CLL-patients and the control group. Comparison of most important HLA alleles at the 6-digit levels between CLL patients and the control group.

| Allele | Cases  *n1* = 29 | Controls  *n2* = 42 | *p*-value | OR | 95% CI | |
| --- | --- | --- | --- | --- | --- | --- |
|  | Number | Number |  |  | Low | Upper |
| HLA-DRB5 01:01:01 | 10 | 17 | 0.76 | 1.12 | 0.530 | 2.374 |
| HLA-DRB5 01:02:01 | 5 | 1 | 0.03 | 0.13 | 0.016 | 1.117 |
| HLA-DRB5 02:02:01 | 14 | 24 | 0.69 | 1.13 | 0.608 | 2.106 |

* Statistical significance was determined after calculating the *p*-value, OR, and CI. The chi-square test or Fisher’s test was used to estimate the differences between the CLL patient and control groups; *n*: number of alleles in the patient and control groups.
