# Supplementary material for: HLA Gene Polymorphisms in Romanian Patients with Chronic Lymphocytic Leukemia
Source: Genet Res (Camb). 2024 Feb 28;2024:8852876. doi: 10.1155/2024/8852876 (PMC10917483; doi:10.1155/2024/8852876)
Supplement: Supplementary Materials — The following supporting information can be downloaded from Supp Table S1: Supplemental Table 1: distribution of HLA alleles in CLL patients and the control group. Comparison of most important HLA alleles at the 6-digit levels between CLL patients and the control group. Supp Table S2: Supplemental Table 2: distribution of HLA-DRB3 in CLL patients and the control group. Comparison of most important HLA alleles at the 6-digit levels between CLL patients and the control group. Supp Table S3: Supplemental Table 3: distribution of HLA-DRB4 in CLL patients and the control group. Comparison of most important HLA alleles at the 6-digit levels between CLL patients and the control group. Supp Table S4: Supplemental Table 4: distribution of HLA-DRB5 in CLL patients and the control group. Comparison of most important HLA alleles at the 6-digit levels between CLL patients and the control group. Supp Table S5: Supplemental Table 5: distribution of HLA alleles in CLL female patients and the female control group. Comparison of most important HLA alleles at the 6-digit levels between CLL women and the women in the control group. Supp Table S6: Supplemental Table 6: distribution of HLA-DRB3 in CLL women patients and the women control group. Comparison of most important HLA alleles at the 6-digit levels between CLL women and the women in the control group. Supp Table S7: Supplemental Table 7: distribution of HLA-DRB4 in CLL women patients and the women control group. Comparison of most important HLA alleles at the 6-digit levels between CLL women and the women in the control group. Supp Table S8: Supplemental Table 8: distribution of HLA-DRB5 in CLL women patients and the women control group. Comparison of most important HLA alleles at the 6-digit levels between CLL women and the women in the control group. Supp Table S9: Supplemental Table 9: distribution of HLA alleles in CLL male patients and the male control group. Comparison of most important HLA alleles at the 6-digit levels bet [file 8852876.f1.zip › Supplemental Table 5 (2).docx]

**Supplemental Table 5.** Distribution of HLA alleles in CLL female patients and the female control group. Comparison of most important HLA alleles at the 6-digit levels between CLL women and the women in the control group.

| Allele | Cases  *n1* = 56 | Controls  *n2* = 92 | *p*-value | OR | 95% CI | |
| --- | --- | --- | --- | --- | --- | --- |
|  | Number | Number |  |  | Low | Upper |
| HLA-A 01:01:01 | 7 | 15 | 0.528 | 1.304 | 0.567 | 3.001 |
| HLA-A 02:01:01 | 17 | 32 | 0.579 | 1.146 | 0.705 | 1.862 |
| HLA-A 02:05:01 | 1 | 0 | 0.378 | 1.018 | 0.983 | 1.055 |
| HLA-A 03:01:01 | 10 | 6 | 0.031 | 0.365 | 00.140 | 0.950 |
| HLA-A 03:02:01 | 2 | 0 | 0.142 | 1.037 | 0.986 | 1.091 |
| HLA-A 11:01:01 | 1 | 3 | 1.000 | 1.826 | 0.195 | 17.130 |
| HLA-A 24:02:01 | 8 | 4 | 0.058 | 0.304 | 0.096 | 0.964 |
| HLA-A 24:03:01 | 1 | 0 | 0.378 | 1.018 | 0.983 | 1.055 |
| HLA-A 25:01:01 | 2 | 1 | 0.557 | 0.304 | 0.028 | 3.280 |
| HLA-A 26:01:01 | 1 | 3 | 1.000 | 1.826 | 0.195 | 17.130 |
| HLA-A 29:01:01 | 0 | 1 | 1.000 | 0.989 | 0.968 | 1.011 |
| HLA-A 29:02:01 | 0 | 2 | 0.526 | 0.978 | 0.949 | 1.009 |
| HLA-A 30:01:01 | 1 | 2 | 1.000 | 1.217 | 0.113 | 13.119 |
| HLA-A 30:04:01 | 0 | 2 | 0.526 | 0.978 | 0.949 | 1.009 |
| HLA-A 32:01:01 | 3 | 6 | 1.000 | 1.217 | 0.317 | 4.675 |
| HLA-A 33:01:01 | 0 | 1 | 1.000 | 0.989 | 0.968 | 1.011 |
| HLA-A 33:03:01 | 0 | 2 | 0.526 | 0.978 | 0.949 | 1.009 |
| HLA-A 66:02:01 | 0 | 1 | 1.000 | 0.989 | 0.968 | 1.011 |
| HLA-A 68:01:02 | 0 | 3 | 0.290 | 0.967 | 0.932 | 1.004 |
| HLA-A 68:02:01 | 1 | 0 | 0.378 | 1.018 | 0.983 | 1.055 |
| HLA-B 07:02:01 | 2 | 5 | 0.710 | 1.522 | 0.305 | 7.581 |
| HLA-B 08:01:01 | 4 | 10 | 0.569 | 1.522 | 0.501 | 4.622 |
| HLA-B 13:02:01 | 0 | 4 | 0.298 | 0.957 | 0.916 | .999 |
| HLA-B 14:02:01 | 0 | 1 | 1.000 | 0.989 | 0.968 | 1.011 |
| HLA-B 15:24:01 | 1 | 0 | 0.378 | 1.018 | 0.983 | 1.055 |
| HLA-B 18:01:01 | 3 | 3 | 0.673 | 0.609 | 0.127 | 2.912 |
| HLA-B 18:03:01 | 0 | 1 | 1.000 | 0.989 | 0.968 | 1.011 |
| HLA-B 18:05:01 | 3 | 1 | 0.152 | 0.203 | 0.022 | 1.903 |
| HLA-B 27:02:01 | 1 | 3 | 1.000 | 1.826 | 0.195 | 17.130 |
| HLA-B 27:05:02 | 1 | 2 | 1.000 | 1.217 | 0.113 | 13.119 |
| HLA-B 35:01:01 | 8 | 3 | 0.021 | 0.228 | 0.063 | 0.825 |
| HLA-B 35:02:01 | 0 | 4 | 0.298 | 0.957 | 0.916 | 0.999 |
| HLA-B 35:03:01 | 4 | 2 | 0.200 | 1.054 | 0.974 | 1.140 |
| HLA-B 37:01:01 | 0 | 2 | 0.526 | 0.978 | 0.949 | 1.009 |
| HLA-B 38:01:01 | 2 | 3 | 1.000 | 0.913 | 0.157 | 5.297 |
| HLA-B 39:01:01 | 4 | 0 | 0.019 | 1.077 | 1.001 | 1.158 |
| HLA-B 39:05:01 | 0 | 1 | 1.000 | 0.989 | 0.968 | 1.011 |
| HLA-B 39:31:01 | 0 | 1 | 1.000 | 0.989 | 0.968 | 1.011 |
| HLA-B 40:01:01 | 0 | 1 | 1.000 | 0.989 | 0.968 | 1.011 |
| HLA-B 40:01:02 | 0 | 1 | 1.000 | 0.989 | 0.968 | 1.011 |
| HLA-B 40:02:01 | 0 | 4 | 0.298 | 0.957 | 0.916 | 0.999 |
| HLA-B 40:06:01 | 0 | 1 | 1.000 | 0.989 | 0.968 | 1.011 |
| HLA-B 41:01:01 | 1 | 2 | 1.000 | 0.818 | 0.072 | 9.236 |
| HLA-B 41:02:01 | 1 | 1 | 1.000 | 0.609 | 0.039 | 9.539 |
| HLA-B 44:02:01 | 2 | 7 | 0.484 | 2.130 | 0.459 | 9.897 |
| HLA-B 44:03:01 | 3 | 5 | 1.000 | 1.014 | 0.252 | 4.082 |
| HLA-B 49:01:01 | 2 | 4 | 1.000 | 1.217 | 0.230 | 6.432 |
| HLA-B 50:01:01 | 1 | 0 | 0.378 | 1.018 | 0.983 | 1.055 |
| HLA-B 51:01:01 | 8 | 9 | 0.405 | 0.685 | 0.280 | 1.672 |
| HLA-B 52:01:01 | 3 | 1 | 0.152 | 0.203 | 0.022 | 1.903 |
| HLA-B 55:01:01 | 2 | 2 | 0.634 | 0.609 | 0.088 | 4.200 |
| HLA-B 56:01:01 | 0 | 2 | 0.526 | 0.978 | 0.949 | 1.009 |
| HLA-B 58:01:01 | 0 | 1 | 1.000 | 0.989 | 0.968 | 1.011 |
| HLA-B 58:02:01 | 0 | 1 | 1.000 | 0.989 | 0.968 | 1.011 |
| HLA-C 01:02:01 | 2 | 3 | 1.000 | 0.913 | 0.157 | 5.297 |
| HLA-C 02:02:02 | 3 | 9 | 0.536 | 1.826 | 0.516 | 6.462 |
| HLA-C 03:02:01 | 0 | 1 | 1.000 | 0.989 | 0.968 | 1.011 |
| HLA-C 03:03:01 | 3 | 1 | 0.152 | 0.203 | 0.022 | 1.903 |
| HLA-C 04:01:01 | 14 | 16 | 0.264 | 0.696 | 0.368 | 1.314 |
| HLA-C 05:01:01 | 1 | 2 | 1.000 | 1.217 | 0.113 | 13.119 |
| HLA-C 06:02:01 | 1 | 7 | 0.259 | 4.261 | 0.538 | 33.726 |
| HLA-C 07:01:01 | 5 | 17 | 0.113 | 2.070 | 0.808 | 5.299 |
| HLA-C 07:02:01 | 5 | 6 | 0.588 | 0.730 | 0.234 | 2.282 |
| HLA-C 07:04:01 | 1 | 3 | 1.000 | 1.826 | 0.195 | 17.130 |
| HLA-C 08:02:01 | 0 | 1 | 1.000 | 0.989 | 0.968 | 1.011 |
| HLA-C 12:02:01 | 2 | 0 | 0.142 | 1.037 | 0.986 | 1.091 |
| HLA-C 12:02:02 | 1 | 3 | 1.000 | 1.826 | 0.195 | 17.130 |
| HLA-C 12:03:01 | 8 | 6 | 0.118 | 0.457 | 0.167 | 1.247 |
| HLA-C 14:02:01 | 1 | 3 | 1.000 | 1.826 | 0.195 | 17.130 |
| HLA-C 15:02:01 | 3 | 7 | 0.743 | 1.420 | 0.383 | 5.269 |
| HLA-C 15:04:01 | 1 | 0 | 0.378 | 1.018 | 0.983 | 1.055 |
| HLA-C 16:01:01 | 1 | 1 | 1.000 | 0.609 | 0.039 | 9.539 |
| HLA-C 16:04:01 | 1 | 2 | 1.000 | 1.217 | 0.113 | 13.119 |
| HLA-C 17:01:01 | 1 | 2 | 1.000 | 1.217 | 0.113 | 13.119 |
| HLA-C 17:03:01 | 1 | 2 | 1.000 | 1.217 | 0.113 | 13.119 |
| HLA-DPA1 01:03:01 | 46 | 67 | 0.196 | 1.716 | 0.753 | 3.912 |
| HLA-DPA1 01:05:01 | 0 | 1 | 1.000 | 0.989 | 0.968 | 1.011 |
| HLA-DPA1 02:01:01 | 6 | 8 | 0.684 | 0.812 | 0.297 | 2.217 |
| HLA-DPA1 02:01:02 | 2 | 9 | 0.208 | 2.739 | 0.614 | 12.224 |
| HLA-DPA1 02:02:02 | 1 | 5 | 0.409 | 3.043 | 0.365 | 25.386 |
| HLA-DPA1 02:07:01 | 0 | 1 | 1.000 | 0.989 | 0.968 | 1.011 |
| HLA-DPA1 03:01:01 | 0 | 1 | 1.000 | 0.989 | 0.968 | 1.011 |
| HLA-DPA1 04:01:01 | 1 | 0 | 0.378 | 1.018 | 0.983 | 1.055 |
| HLA-DPB1 01:01:01 | 3 | 9 | 0.536 | 1.826 | 0.516 | 6.462 |
| HLA-DPB1 02:01:02 | 6 | 11 | 1.000 | 1.116 | 0.437 | 2.850 |
| HLA-DPB1 02:02:01 | 0 | 1 | 1.000 | 0.989 | 0.968 | 1.011 |
| HLA-DPB1 03:01:01 | 6 | 6 | 0.365 | 0.609 | 0.206 | 1.796 |
| HLA-DPB1 04:01:01 | 20 | 31 | 0.859 | 0.943 | 0.600 | 1.485 |
| HLA-DPB1 04:02:01 | 11 | 20 | 0.761 | 1.107 | 0.574 | 2.134 |
| HLA-DPB1 05:01:01 | 1 | 4 | 0.650 | 2.435 | 0.279 | 21.239 |
| HLA-DPB1 06:01:01 | 0 | 1 | 1.000 | 0.989 | 0.968 | 1.011 |
| HLA-DPB1 09:01:01 | 0 | 1 | 1.000 | 0.989 | 0.968 | 1.011 |
| HLA-DPB1 10:01:01 | 2 | 2 | 0.634 | 0.609 | 0.088 | 4.200 |
| HLA-DPB1 11:01:01 | 1 | 0 | 0.378 | 1.018 | 0.983 | 1.055 |
| HLA-DPB1 13:01:01 | 0 | 1 | 1.000 | 0.989 | 0.968 | 1.011 |
| HLA-DPB1 14:01:01 | 1 | 0 | 0.378 | 1.018 | 0.983 | 1.055 |
| HLA-DPB1 17:01:01 | 1 | 2 | 1.000 | 1.217 | 0.113 | 13.119 |
| HLA-DPB1 18:01:01 | 0 | 1 | 1.000 | 0.989 | 0.968 | 1.011 |
| HLA-DPB1 28:01:01 | 1 | 0 | 0.378 | 1.018 | 0.983 | 1.055 |
| HLA-DPB1 104:01:01 | 0 | 1 | 1.000 | 0.989 | 0.968 | 1.011 |
| HLA-DPB1 105:01:01 | 0 | 1 | 1.000 | 0.989 | 0.968 | 1.011 |
| HLA-DQA1 01:01:01 | 4 | 10 | 0.569 | 1.522 | 0.501 | 4.622 |
| HLA-DQA1 01:02:01 | 8 | 22 | 0.158 | 1.674 | 0.800 | 3.501 |
| HLA-DQA1 01:02:02 | 3 | 7 | 0.743 | 1.420 | 0.383 | 5.269 |
| HLA-DQA1 01:03:01 | 4 | 5 | 0.730 | 0.761 | 0.213 | 2.715 |
| HLA-DQA1 01:04:01 | 3 | 3 | 0.673 | 0.609 | 0.127 | 2.912 |
| HLA-DQA1 01:04:02 | 0 | 1 | 1.000 | 0.989 | 0.968 | 1.011 |
| HLA-DQA1 01:05:01 | 1 | 0 | 0.378 | 1.018 | 0.983 | 1.055 |
| HLA-DQA1 02:01:01 | 4 | 6 | 1.000 | 0.913 | 0.269 | 3.095 |
| HLA-DQA1 03:01:01 | 4 | 5 | 0.730 | 0.761 | 0.213 | 2.715 |
| HLA-DQA1 03:03:01 | 0 | 1 | 1.000 | 0.989 | 0.968 | 1.011 |
| HLA-DQA1 04:01:01 | 0 | 1 | 1.000 | 0.989 | 0.968 | 1.011 |
| HLA-DQA1 05:01:01 | 9 | 19 | 0.490 | 1.285 | 0.625 | 2.640 |
| HLA-DQA1 05:03:01 | 1 | 0 | 0.378 | 1.018 | 0.983 | 1.055 |
| HLA-DQA1 05:05:01 | 15 | 12 | 0.036 | 0.487 | 0.246 | 0.964 |
| HLA-DQB1 02:01:01 | 8 | 13 | 0.979 | 0.989 | 0.438 | 2.236 |
| HLA-DQB1 02:02:01 | 2 | 5 | 0.710 | 1.522 | 0.305 | 7.581 |
| HLA-DQB1 03:01:01 | 14 | 18 | 0.436 | 0.783 | 0.423 | 1.447 |
| HLA-DQB1 03:02:01 | 4 | 2 | 0.200 | 0.304 | 0.058 | 1.608 |
| HLA-DQB1 03:03:02 | 0 | 1 | 1.000 | 0.989 | 0.968 | 1.011 |
| HLA-DQB1 03:04:01 | 0 | 1 | 1.000 | 0.989 | 0.968 | 1.011 |
| HLA-DQB1 03:19:01 | 0 | 1 | 1.000 | 0.989 | 0.968 | 1.011 |
| HLA-DQB1 04:02:01 | 0 | 1 | 1.000 | 0.989 | 0.968 | 1.011 |
| HLA-DQB1 05:01:01 | 5 | 8 | 0.961 | 0.974 | 0.335 | 2.830 |
| HLA-DQB1 05:02:01 | 7 | 17 | 0.339 | 1.478 | 0.654 | 3.340 |
| HLA-DQB1 05:03:01 | 3 | 6 | 1.000 | 1.217 | 0.317 | 4.675 |
| HLA-DQB1 05:04:01 | 0 | 1 | 1.000 | 0.989 | 0.968 | 1.011 |
| HLA-DQB1 06:01:01 | 2 | 1 | 0.557 | 0.304 | 0.028 | 3.280 |
| HLA-DQB1 06:02:01 | 6 | 8 | 0.684 | 0.812 | 0.297 | 2.217 |
| HLA-DQB1 06:03:01 | 3 | 4 | 1.000 | 0.812 | 0.189 | 3.493 |
| HLA-DQB1 06:03:11 | 0 | 1 | 1.000 | 0.989 | 0.968 | 1.011 |
| HLA-DQB1 06:04:01 | 2 | 3 | 1.000 | 0.913 | 0.157 | 5.297 |
| HLA-DQB1 06:09:01 | 0 | 1 | 1.000 | 0.989 | 0.968 | 1.011 |
| HLA-DRB1 01:01:01 | 4 | 7 | 1.000 | 1.065 | 0.326 | 3.476 |
| HLA-DRB1 03:01:01 | 7 | 12 | 0.924 | 1.043 | 0.437 | 2.493 |
| HLA-DRB1 04:01:01 | 1 | 0 | 0.378 | 1.018 | 0.983 | 1.055 |
| HLA-DRB1 04:02:01 | 2 | 0 | 0.142 | 1.037 | 0.986 | 1.091 |
| HLA-DRB1 04:03:01 | 0 | 2 | 0.526 | 0.978 | 0.949 | 1.009 |
| HLA-DRB1 04:04:01 | 0 | 1 | 1.000 | 0.989 | 0.968 | 1.011 |
| HLA-DRB1 04:05:01 | 1 | 0 | 0.378 | 1.018 | 0.983 | 1.055 |
| HLA-DRB1 07:01:01 | 3 | 13 | 0.110 | 2.638 | 0.786 | 8.851 |
| HLA-DRB1 08:01:01 | 0 | 1 | 1.000 | 0.989 | 0.968 | 1.011 |
| HLA-DRB1 10:01:01 | 1 | 1 | 1.000 | 0.609 | 0.039 | 9.539 |
| HLA-DRB1 11:01:01 | 8 | 6 | 0.118 | 0.457 | 0.167 | 1.247 |
| HLA-DRB1 11:02:01 | 0 | 1 | 1.000 | 0.989 | 0.968 | 1.011 |
| HLA-DRB1 11:03:01 | 0 | 2 | 0.526 | 0.978 | 0.949 | 1.009 |
| HLA-DRB1 11:04:01 | 2 | 7 | 0.484 | 2.130 | 0.459 | 9.897 |
| HLA-DRB1 12:01:01 | 2 | 0 | 0.142 | 1.037 | 0.986 | 1.091 |
| HLA-DRB1 13:01:01 | 3 | 6 | 1.000 | 1.217 | 0.317 | 4.675 |
| HLA-DRB1 13:02:01 | 2 | 5 | 0.710 | 1.522 | 0.305 | 7.581 |
| HLA-DRB1 13:03:01 | 2 | 1 | 0.557 | 0.304 | 0.028 | 3.280 |
| HLA-DRB1 13:05:01 | 0 | 1 | 1.000 | 0.989 | 0.968 | 1.011 |
| HLA-DRB1 14:01:01 | 3 | 3 | 0.673 | 0.609 | 0.127 | 2.912 |
| HLA-DRB1 14:04:01 | 0 | 1 | 1.000 | 0.989 | 0.968 | 1.011 |
| HLA-DRB1 14:54:01 | 0 | 2 | 0.526 | 0.978 | 0.949 | 1.009 |
| HLA-DRB1 15:01:01 | 5 | 6 | 0.588 | 0.730 | 0.234 | 2.282 |
| HLA-DRB1 15:02:01 | 3 | 1 | 0.152 | 0.203 | 0.022 | 1.903 |
| HLA-DRB1 16:01:01 | 7 | 10 | 0.794 | 0.870 | 0.351 | 2.154 |
| HLA-DRB1 16:02:01 | 0 | 2 | 0.526 | 0.978 | 0.949 | 1.009 |

* Statistical significance was determined after calculating the *p*-value, OR, and CI. The chi-square test or Fisher’s test was used to estimate the differences between the CLL patient and control groups; *n*: number of alleles in the patient and control groups.
