# Supplementary material for: HLA Gene Polymorphisms in Romanian Patients with Chronic Lymphocytic Leukemia
Source: Genet Res (Camb). 2024 Feb 28;2024:8852876. doi: 10.1155/2024/8852876 (PMC10917483; doi:10.1155/2024/8852876)
Supplement: Supplementary Materials — The following supporting information can be downloaded from Supp Table S1: Supplemental Table 1: distribution of HLA alleles in CLL patients and the control group. Comparison of most important HLA alleles at the 6-digit levels between CLL patients and the control group. Supp Table S2: Supplemental Table 2: distribution of HLA-DRB3 in CLL patients and the control group. Comparison of most important HLA alleles at the 6-digit levels between CLL patients and the control group. Supp Table S3: Supplemental Table 3: distribution of HLA-DRB4 in CLL patients and the control group. Comparison of most important HLA alleles at the 6-digit levels between CLL patients and the control group. Supp Table S4: Supplemental Table 4: distribution of HLA-DRB5 in CLL patients and the control group. Comparison of most important HLA alleles at the 6-digit levels between CLL patients and the control group. Supp Table S5: Supplemental Table 5: distribution of HLA alleles in CLL female patients and the female control group. Comparison of most important HLA alleles at the 6-digit levels between CLL women and the women in the control group. Supp Table S6: Supplemental Table 6: distribution of HLA-DRB3 in CLL women patients and the women control group. Comparison of most important HLA alleles at the 6-digit levels between CLL women and the women in the control group. Supp Table S7: Supplemental Table 7: distribution of HLA-DRB4 in CLL women patients and the women control group. Comparison of most important HLA alleles at the 6-digit levels between CLL women and the women in the control group. Supp Table S8: Supplemental Table 8: distribution of HLA-DRB5 in CLL women patients and the women control group. Comparison of most important HLA alleles at the 6-digit levels between CLL women and the women in the control group. Supp Table S9: Supplemental Table 9: distribution of HLA alleles in CLL male patients and the male control group. Comparison of most important HLA alleles at the 6-digit levels bet [file 8852876.f1.zip › Supplemental Table 9 (2).docx]

**Supplemental Table 9.** Distribution of HLA alleles in CLL male patients and the male control group. Comparison of most important HLA alleles at the 6-digit levels between CLL men and the men in the control group.

| Allele | Cases  *n1* = 76 | Controls  *n2* = 108 | *p*-value | OR | 95% CI | |
| --- | --- | --- | --- | --- | --- | --- |
|  | Number | Number |  |  | Low | Upper |
| HLA-A 01:01:01 | 7 | 13 | 0.544 | 1.307 | .547 | 3.122 |
| HLA-A 02:01:01 | 21 | 27 | 0.689 | 0.905 | 0.555 | 1.475 |
| HLA-A 02:02:01 | 0 | 2 | 0.512 | 0.981 | 0.956 | 1.007 |
| HLA-A 02:05:01 | 2 | 1 | 0.570 | 0.352 | 0.032 | 3.811 |
| HLA-A 03:01:01 | 8 | 14 | 0.616 | 1.231 | 0.544 | 2.789 |
| HLA-A 03:02:01 | 1 | 0 | 0.413 | 1.013 | 0.987 | 1.040 |
| HLA-A 11:01:01 | 3 | 5 | 1.000 | 1.173 | 0.289 | 4.761 |
| HLA-A 24:02:01 | 9 | 6 | 0.125 | 0.469 | 0.174 | 1.263 |
| HLA-A 24:03:01 | 1 | 0 | 0.413 | 1.013 | .987 | 1.040 |
| HLA-A 25:01:01 | 4 | 4 | 0.719 | 0.704 | 0.182 | 2.727 |
| HLA-A 26:01:01 | 4 | 5 | 1.000 | 0.880 | 0.244 | 3.169 |
| HLA-A 29:01:01 | 1 | 0 | 0.413 | 1.013 | 0.987 | 1.040 |
| HLA-A 29:02:01 | 1 | 2 | 1.000 | 1.407 | 0.130 | 15.244 |
| HLA-A 30:01:01 | 0 | 6 | 0.043 | 0.944 | 0.902 | 0.989 |
| HLA-A 30:04:01 | 0 | 1 | 1.000 | 0.991 | 0.973 | 1.009 |
| HLA-A 31:01:02 | 0 | 3 | 0.269 | 0.972 | 0.942 | 1.004 |
| HLA-A 32:01:01 | 4 | 8 | 0.764 | 1.407 | 0.440 | 4.507 |
| HLA-A 33:01:01 | 1 | 0 | 0.413 | 1.013 | 0.987 | 1.040 |
| HLA-A 33:01:02 | 1 | 0 | 0.413 | 1.013 | 0.987 | 1.040 |
| HLA-A 33:03:01 | 0 | 1 | 1.000 | 0.991 | 0.973 | 1.009 |
| HLA-A 66:01:01 | 1 | 0 | 0.413 | 1.013 | 0.987 | 1.040 |
| HLA-A 68:01:01 | 1 | 3 | 0.644 | 2.111 | 0.224 | 19.911 |
| HLA-A 68:02:01 | 1 | 0 | 0.413 | 1.013 | 0.987 | 1.040 |
| HLA-B 07:01:01 | 1 | 0 | 0.413 | 1.013 | 0.987 | 1.040 |
| HLA-B 07:02:01 | 4 | 4 | 0.719 | 0.704 | 0.182 | 2.727 |
| HLA-B 07:05:01 | 1 | 0 | 0.413 | 1.013 | 0.987 | 1.040 |
| HLA-B 08:01:01 | 5 | 9 | 0.659 | 1.267 | 0.442 | 3.631 |
| HLA-B 13:02:01 | 4 | 6 | 1.000 | 1.056 | 0.308 | 3.614 |
| HLA-B 14:01:01 | 0 | 1 | 1.000 | 0.991 | 0.973 | 1.009 |
| HLA-B 14:02:01 | 2 | 2 | 1.000 | 0.702 | 0.101 | 4.886 |
| HLA-B 15:01:01 | 2 | 2 | 1.000 | 0.702 | 0.101 | 4.886 |
| HLA-B 15:03:01 | 0 | 1 | 1.000 | 0.991 | 0.973 | 1.009 |
| HLA-B 15:10:01 | 0 | 1 | 1.000 | 0.991 | 0.973 | 1.009 |
| HLA-B 18:01:01 | 12 | 14 | 0.588 | 0.821 | 0.402 | 1.675 |
| HLA-B 18:04:01 | 0 | 1 | 1.000 | 0.991 | 0.973 | 1.009 |
| HLA-B 18:05:01 | 2 | 1 | 0.570 | 0.352 | 0.032 | 3.811 |
| HLA-B 27:02:01 | 2 | 2 | 1.000 | 0.702 | 0.101 | 4.886 |
| HLA-B 27:05:02 | 2 | 2 | 1.000 | 0.702 | 0.101 | 4.886 |
| HLA-B 35:01:01 | 2 | 3 | 1.000 | 1.056 | 0.181 | 6.166 |
| HLA-B 35:02:01 | 2 | 3 | 1.000 | 1.056 | 0.181 | 6.166 |
| HLA-B 35:03:01 | 3 | 4 | 1.000 | 0.938 | 0.216 | 4.072 |
| HLA-B 35:08:01 | 0 | 1 | 1.000 | 0.991 | 0.973 | 1.009 |
| HLA-B 37:01:01 | 0 | 1 | 1.000 | 0.991 | 0.973 | 1.009 |
| HLA-B 38:01:01 | 6 | 3 | 0.165 | 0.352 | 0.091 | 1.363 |
| HLA-B 39:01:01 | 1 | 2 | 1.000 | 1.407 | 0.130 | 15.244 |
| HLA-B 40:01:01 | 0 | 1 | 1.000 | 0.991 | 0.973 | 1.009 |
| HLA-B 40:01:02 | 0 | 1 | 1.000 | 0.991 | 0.973 | 1.009 |
| HLA-B 40:02:01 | 0 | 2 | 0.512 | 0.981 | 0.956 | 1.007 |
| HLA-B 41:01:01 | 1 | 3 | 0.644 | 2.111 | 0.224 | 19.911 |
| HLA-B 41:02:01 | 2 | 2 | 1.000 | 0.702 | 0.101 | 4.886 |
| HLA-B 44:02:01 | 4 | 4 | 0.719 | 0.704 | 0.182 | 2.727 |
| HLA-B 44:03:01 | 5 | 3 | 0.278 | 0.422 | 0.104 | 1.714 |
| HLA-B 49:01:01 | 2 | 1 | 0.570 | 0.352 | 0.032 | 3.811 |
| HLA-B 50:01:01 | 2 | 0 | 0.169 | 1.027 | 0.990 | 1.066 |
| HLA-B 51:01:01 | 3 | 14 | 0.041 | 3.284 | 0.977 | 11.034 |
| HLA-B 51:05:01 | 1 | 0 | 0.413 | 1.013 | 0.987 | 1.040 |
| HLA-B 52:01:01 | 1 | 3 | 0.644 | 2.111 | 0.224 | 19.911 |
| HLA-B 55:01:01 | 2 | 3 | 1.000 | 1.056 | 0.181 | 6.166 |
| HLA-B 58:01:01 | 1 | 1 | 1.000 | 0.704 | 0.045 | 11.077 |
| HLA-B 59:01:01 | 0 | 1 | 1.000 | 0.991 | 0.973 | 1.009 |
| HLA-B 81:01:01 | 0 | 1 | 1.000 | 0.991 | 0.973 | 1.009 |
| HLA-C 01:02:01 | 3 | 8 | 0.530 | 1.877 | 0.514 | 6.845 |
| HLA-C 02:02:02 | 2 | 7 | 0.311 | 2.463 | 0.526 | 11.533 |
| HLA-C 03:02:01 | 1 | 2 | 1.000 | 1.407 | 0.130 | 15.244 |
| HLA-C 03:02:02 | 0 | 1 | 1.000 | 0.991 | 0.973 | 1.009 |
| HLA-C 03:03:01 | 3 | 2 | 0.406 | 0.469 | 0.080 | 2.740 |
| HLA-C 03:04:01 | 1 | 4 | 0.650 | 2.815 | 0.321 | 24.692 |
| HLA-C 03:04:02 | 0 | 1 | 1.000 | 0.991 | 0.973 | 1.009 |
| HLA-C 04:01:01 | 12 | 13 | 0.516 | 0.762 | 0.368 | 1.578 |
| HLA-C 05:01:01 | 1 | 2 | 1.000 | 1.407 | 0.130 | 15.244 |
| HLA-C 06:02:01 | 5 | 8 | 0.829 | 1.126 | 0.383 | 3.309 |
| HLA-C 07:01:01 | 16 | 16 | 0.272 | 0.704 | 0.376 | 1.318 |
| HLA-C 07:02:01 | 5 | 5 | 0.566 | 0.704 | 0.211 | 2.347 |
| HLA-C 08:02:01 | 2 | 3 | 1.000 | 1.056 | 0.181 | 6.166 |
| HLA-C 12:02:01 | 1 | 0 | 0.413 | 1.013 | 0.987 | 1.040 |
| HLA-C 12:02:02 | 0 | 3 | 0.269 | 0.972 | 0.942 | 1.004 |
| HLA-C 12:03:01 | 11 | 16 | 0.949 | 1.024 | 0.504 | 2.081 |
| HLA-C 12:12:01 | 0 | 1 | 1.000 | 0.991 | 0.973 | 1.009 |
| HLA-C 14:02:01 | 1 | 0 | 0.413 | 1.013 | 0.987 | 1.040 |
| HLA-C 15:02:01 | 1 | 1 | 1.000 | 0.704 | 0.045 | 11.077 |
| HLA-C 15:05:01 | 2 | 0 | 0.169 | 1.027 | 0.990 | 1.066 |
| HLA-C 15:13:01 | 0 | 2 | 0.512 | 0.981 | 0.956 | 1.007 |
| HLA-C 16:01:01 | 1 | 3 | 0.644 | 2.111 | 0.224 | 19.911 |
| HLA-C 16:02:01 | 0 | 2 | 0.512 | 0.981 | 0.956 | 1.007 |
| HLA-C 16:04:01 | 3 | 1 | 0.308 | 0.235 | 0.025 | 2.212 |
| HLA-C 17:01:01 | 1 | 2 | 1.000 | 1.407 | 0.130 | 15.244 |
| HLA-C 17:03:01 | 2 | 2 | 1.000 | 0.702 | 0.101 | 4.886 |
| HLA-C 18:01:01 | 0 | 1 | 1.000 | 0.991 | 0.973 | 1.009 |
| HLA-DPA1 01:03:01 | 62 | 85 | 0.632 | 0.965 | 0.834 | 1.115 |
| HLA-DPA1 02:01:01 | 10 | 14 | 0.969 | 0.985 | 0.462 | 2.099 |
| HLA-DPA1 02:01:02 | 3 | 4 | 1.000 | 0.938 | 0.216 | 4.072 |
| HLA-DPA1 02:02:02 | 0 | 5 | 0.078 | 0.954 | 0.915 | 0.994 |
| HLA-DPA1 02:04:01 | 1 | 0 | 0.413 | 1.013 | 0.987 | 1.040 |
| HLA-DPB1 01:01:01 | 2 | 7 | 0.311 | 2.463 | 0.526 | 11.533 |
| HLA-DPB1 02:01:02 | 18 | 20 | 0.460 | 0.782 | 0.444 | 1.376 |
| HLA-DPB1 03:01:01 | 5 | 9 | 0.659 | 1.267 | 0.442 | 3.631 |
| HLA-DPB1 04:01:01 | 25 | 38 | 0.747 | 1.070 | 0.709 | 1.613 |
| HLA-DPB1 04:02:01 | 13 | 14 | 0.434 | 0.758 | 0.378 | 1.519 |
| HLA-DPB1 05:01:01 | 1 | 3 | 0.644 | 2.111 | 0.224 | 19.911 |
| HLA-DPB1 09:01:01 | 1 | 2 | 1.000 | 1.407 | 0.130 | 15.244 |
| HLA-DPB1 10:01:01 | 2 | 4 | 1.000 | 1.407 | 0.264 | 7.490 |
| HLA-DPB1 11:01:01 | 1 | 0 | 0.413 | 1.013 | 0.987 | 1.040 |
| HLA-DPB1 13:01:01 | 2 | 0 | 0.162 | 1.027 | 0.990 | 1.066 |
| HLA-DPB1 14:01:01 | 1 | 1 | 1.000 | 0.704 | 0.045 | 11.077 |
| HLA-DPB1 15:01:01 | 0 | 1 | 1.000 | 0.991 | 0.973 | 1.009 |
| HLA-DPB1 17:01:01 | 1 | 5 | 0.403 | 3.519 | 0.419 | 29.516 |
| HLA-DPB1 23:01:01 | 1 | 1 | 1.000 | 0.704 | 0.045 | 11.077 |
| HLA-DPB1 28:01:01 | 1 | 0 | 0.413 | 1.013 | 0.987 | 1.040 |
| HLA-DPB1 104:01:01 | 1 | 2 | 1.000 | 1.407 | 0.130 | 15.244 |
| HLA-DQA1 01:01:01 | 3 | 9 | 0.364 | 2.111 | 0.591 | 7.542 |
| HLA-DQA1 01:02:01 | 7 | 16 | 0.366 | 1.608 | 0.696 | 3.719 |
| HLA-DQA1 01:02:02 | 3 | 11 | 0.160 | 2.580 | 0.745 | 8.938 |
| HLA-DQA1 01:03:01 | 6 | 6 | 0.527 | 0.704 | 0.236 | 2.099 |
| HLA-DQA1 01:04:01 | 2 | 2 | 1.000 | 0.704 | 0.101 | 4.886 |
| HLA-DQA1 01:05:01 | 2 | 2 | 1.000 | 0.704 | 0.101 | 4.886 |
| HLA-DQA1 01:05:02 | 0 | 1 | 1.000 | 0.991 | 0.973 | 1.009 |
| HLA-DQA1 02:01:01 | 10 | 14 | 1.000 | 0.985 | 0.462 | 2.099 |
| HLA-DQA1 03:01:01 | 11 | 7 | 0.072 | 0.448 | 0.182 | 1.103 |
| HLA-DQA1 03:02:01 | 1 | 0 | 0.413 | 1.013 | 0.987 | 1.040 |
| HLA-DQA1 03:03:01 | 2 | 0 | 0.169 | 1.027 | 0.990 | 1.066 |
| HLA-DQA1 04:01:01 | 1 | 2 | 1.000 | 1.407 | 0.130 | 15.244 |
| HLA-DQA1 05:01:01 | 13 | 24 | 0.394 | 1.299 | 0.707 | 2.386 |
| HLA-DQA1 05:05:01 | 14 | 12 | 0.161 | 0.603 | 0.296 | 1.231 |
| HLA-DQA1 05:09:01 | 0 | 1 | 1.000 | 0.991 | 0.973 | 1.009 |
| HLA-DQA1 06:01:01 | 1 | 1 | 1.000 | 0.704 | 0.045 | 11.077 |
| HLA-DQB1 02:01:01 | 8 | 108 | 0.239 | 1.583 | 0.726 | 3.452 |
| HLA-DQB1 02:02:01 | 8 | 8 | 0.460 | 0.704 | 0.276 | 1.793 |
| HLA-DQB1 03:01:01 | 18 | 26 | 0.951 | 1.016 | 0.602 | 1.717 |
| HLA-DQB1 03:02:01 | 9 | 6 | 0.125 | 0.469 | 0.174 | 1.263 |
| HLA-DQB1 03:03:02 | 1 | 1 | 1.000 | 0.704 | 0.045 | 11.077 |
| HLA-DQB1 04:02:01 | 1 | 2 | 1.000 | 1.407 | 0.130 | 15.244 |
| HLA-DQB1 05:01:01 | 5 | 11 | 0.393 | 1.548 | 0.561 | 4.274 |
| HLA-DQB1 05:02:01 | 9 | 17 | 0.455 | 1.329 | 0.626 | 2.822 |
| HLA-DQB1 05:03:01 | 4 | 2 | 0.232 | 0.352 | 0.066 | 1.872 |
| HLA-DQB1 06:01:01 | 3 | 1 | 0.308 | 0.235 | 0.025 | 2.212 |
| HLA-DQB1 06:02:01 | 4 | 7 | 1.000 | 1.231 | 0.374 | 4.060 |
| HLA-DQB1 06:03:01 | 4 | 6 | 1.000 | 1.056 | 0.308 | 3.614 |
| HLA-DQB1 06:04:01 | 0 | 2 | 0.512 | 0.981 | 0.956 | 1.007 |
| HLA-DQB1 06:09:01 | 0 | 1 | 1.000 | 0.991 | 0.973 | 1.009 |
| HLA-DRB1 01:01:01 | 2 | 8 | 0.200 | 2.815 | 0.615 | 12.889 |
| HLA-DRB1 01:02:01 | 0 | 1 | 1.000 | 0.991 | 0.973 | 1.009 |
| HLA-DRB1 03:01:01 | 5 | 13 | 0.220 | 1.830 | 0.681 | 4.918 |
| HLA-DRB1 03:02:01 | 0 | 1 | 1.000 | 0.991 | 0.973 | 1.009 |
| HLA-DRB1 04:01:01 | 1 | 4 | 0.650 | 2.815 | 0.321 | 24.692 |
| HLA-DRB1 04:02:01 | 5 | 0 | 0.011 | 1.070 | 1.008 | 1.136 |
| HLA-DRB1 04:03:01 | 3 | 0 | 0.069 | 1.041 | 0.995 | 1.090 |
| HLA-DRB1 04:04:01 | 0 | 1 | 1.000 | 0.991 | 0.973 | 1.009 |
| HLA-DRB1 04:05:01 | 2 | 1 | 0.570 | 0.352 | 0.032 | 3.811 |
| HLA-DRB1 04:07:01 | 1 | 0 | 0.413 | 1.013 | 0.987 | 1.040 |
| HLA-DRB1 07:01:01 | 9 | 15 | 0.684 | 1.173 | 0.542 | 2.539 |
| HLA-DRB1 08:01:01 | 1 | 1 | 1.000 | 0.704 | 0.045 | 11.077 |
| HLA-DRB1 09:01:02 | 1 | 0 | 0.413 | 1.013 | 0.987 | 1.040 |
| HLA-DRB1 10:01:01 | 2 | 2 | 1.000 | 0.704 | 0.101 | 4.886 |
| HLA-DRB1 11:01:01 | 3 | 1 | 0.308 | 0.235 | 0.025 | 2.212 |
| HLA-DRB1 11:02:01 | 0 | 1 | 1.000 | 0.991 | 0.973 | 1.009 |
| HLA-DRB1 11:03:01 | 0 | 1 | 1.000 | 0.991 | 0.973 | 1.009 |
| HLA-DRB1 11:04:01 | 11 | 15 | 0.911 | 0.960 | 0.467 | 1.973 |
| HLA-DRB1 12:01:01 | 0 | 4 | 0.144 | 0.963 | 0.928 | 0.999 |
| HLA-DRB1 13:01:01 | 4 | 5 | 1.000 | 0.880 | 0.244 | 3.169 |
| HLA-DRB1 13:02:01 | 0 | 4 | 0.144 | 0.963 | 0.928 | 0.999 |
| HLA-DRB1 13:03:01 | 3 | 4 | 1.000 | 0.938 | 0.216 | 4.072 |
| HLA-DRB1 14:01:01 | 3 | 0 | 0.069 | 1.041 | 0.995 | 1.090 |
| HLA-DRB1 14:04:01 | 1 | 0 | 0.413 | 1.013 | 0.987 | 1.040 |
| HLA-DRB1 14:54:01 | 0 | 3 | 0.269 | 0.972 | 0.942 | 1.004 |
| HLA-DRB1 15:01:01 | 6 | 8 | 1.000 | 0.938 | 0.339 | 2.594 |
| HLA-DRB1 15:02:01 | 3 | 1 | 0.406 | 0.469 | 0.080 | 2.740 |
| HLA-DRB1 16:01:01 | 9 | 10 | 0.627 | 0.782 | 0.334 | 1.832 |
| HLA-DRB1 16:02:01 | 0 | 2 | 0.512 | 0.981 | 0.956 | 1.007 |

* Statistical significance was determined after calculating the *p*-value, OR, and CI. The chi-square test or Fisher’s test was used to estimate the differences between the CLL patient and control groups; *n*: number of alleles in the patient and control groups.
